# Comparative transcriptome analysis of trout skin pigment cells

**DOI:** 10.1186/s12864-019-5714-1

**Published:** 2019-05-09

**Authors:** Ida Djurdjevič, Tomasz Furmanek, Seita Miyazawa, Simona Sušnik Bajec

**Affiliations:** 1 Department of Animal Science, Biotechnical Faculty, University of Ljubljana, Groblje 3, SI-1230 Domžale, Slovenia; 2 Institute of Marine Research, Bergen, Norway; 3 Graduate School of Frontier Biosciences, Osaka University, Osaka, Japan

**Keywords:** *Salmo marmoratus*, *Salmo trutta*, Pigment cell, DEG, RNA-seq, qPCR

## Abstract

**Background:**

Enormous variability in skin colour and patterning is a characteristic of teleost fish, including Salmonidae fishes, which present themselves as a suitable model for studying mechanisms of pigment patterning. In order to screen for candidate genes potentially involved in the specific skin pigment pattern in marble trout (labyrinthine skin pattern) and brown trout (spotted skin pattern), we conducted comparative transcriptome analysis between differently pigmented dermis sections of the adult skin of the two species.

**Results:**

Differentially expressed genes (DEGs) possibly associated with skin pigment pattern were identified. The expression profile of 27 DEGs was further tested with quantitative real-time PCR on a larger number of samples. Expression of a subset of ten of these genes was analysed in hybrid (marble x brown) trout individuals and compared with the complexity of their skin pigment pattern. A correlation between the phenotype and the expression profile assessed for hybrid individuals was detected for four (*gja5*, *clcn2*, *cdkn1a* and *tjp1*) of the ten candidate genes tested. The potential role of these genes in skin pigment pattern maintenance is discussed.

**Conclusions:**

Our results indicate that the maintenance of different pigment patterns in trout is dependent upon specific communication—involving gap junctions, tight junctions and ion channels—between chromatophores present in differentially pigmented skin regions.

**Electronic supplementary material:**

The online version of this article (10.1186/s12864-019-5714-1) contains supplementary material, which is available to authorized users.

## Background

Skin pigmentation and pigment patterning play an important role in the survival of animals. Pigments can influence thermoregulation, social communication, mate choice and predator avoidance, among others, and provide protection against the harmful effect of solar radiation [1, 2]. Even closely related animals exhibit extremely divergent patterns indicative of great plasticity in skin patterning [3]. Enormous variability in skin colour and pattern is characteristic of teleost fish, in which there are six known pigment cell types: melanophores, iridophores, xanthophores, erythrophores, leucophores and cyanophores [4, 5]. These chromatophores synthesise and retain their specific pigments and inner structures intracellularly [6]. Skin pigmentation and pattern thus reflect the numbers and arrangements of the chromatophores themselves. Although only one pigment cell type is known in mammals, there is much conservation of pigmentation-related genes in teleosts and mammals [7]. Therefore, given also the great variability created by numerous pigment cell types, teleosts are important models for studying the cellular, physiological, genetic and environmental factors involved in pigment pattern formation and maintenance [2, 8, 9].

The genetic basis of pigment production and subsequent skin pigmentation is well understood in model species such as zebrafish [10–12], and some mechanisms regulating pigment patterning in zebrafish have been recently proposed (e.g. [13–15]). Many studies conducted on zebrafish show a requirement for interactions among three types of pigment cell—melanophore, xanthophore and iridophore—for stripe formation in adult fish [16–19] and the importance of cell–cell communication in the formation and maintenance of different pigment patterns. Through induction of single gene mutations, important roles in colour patterning have been identified in ion channels [20], tight junctions [21] and gap junctions [16, 22, 23]. The results of those studies show that change in bioelectrical communication between pigment cells can greatly affect skin pigment pattern [24]. Along with chemical diffusion bioelectric signalling mechanisms are fundamental properties for pattern self-generation, as envisaged in the Turing theoretical model for morphogenesis in living systems [24–26].

Much research on zebrafish has been undertaken on artificially produced mutants. However, much less is known about the genetics of skin pigment patterns in natural populations. Salmonidae fishes are characterised by a huge variety of naturally present pigment patterns and thus present themselves as a suitable model for studying the mechanism of pigment pattern formation and maintenance.

One factor that may cause great variety in salmonids could be the relatively recent whole genome duplication (WGD) event (salmonid-specific 4th WGD, or Ss4R [27],) that took place some 25–100 million years ago. Analysis of the rainbow trout genome [28] has shown that half (48%) of the protein-coding genes retain both ohnologues (paralogues formed by a WGD event). Several instances of gene duplication in pigment pathways have also been observed [7, 29, 30] and may have led to diversity of skin colour and patterns in this group of fishes.

**Fig. 1:**
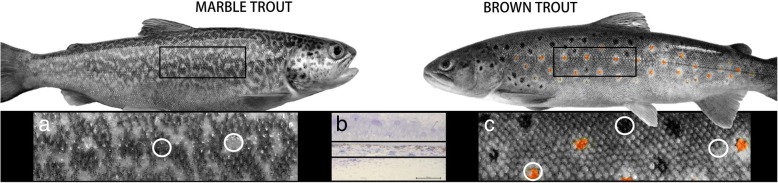
Marble and brown trout and their typical skin pigment pattern: **a**, Labyrinthine pattern on the skin of marble trout; **b**, The part of the dermis used for preparing cryosections; Scale bar: 50 μm; **c**, Spot pattern on the skin of brown trout. Circles in **a** and **c** highlight the differently pigmented parts dissected with biopsy punches

In the present study, we focused on a very closely related [31], yet phenotypically divergent, sister taxon pair in the genus *Salmo*: marble trout (*S. marmoratus*), with labyrinthine skin pattern, and brown trout (*S. trutta*), with spotted skin pattern (Fig. 1). Changes in skin pigmentation and pattern can occur over surprisingly short evolutionary timescales, as in these *Salmo* species, while the reoccurrence of similar colour patterns across large phylogenetic distances is common. A labyrinthine pattern is found in pufferfish (*Takifugu exascurus*), some zebrafish mutants (Cx41.8 M7 [3]), partially in jaguar/obelix [20], and also in an isolated population of brown trout inhabiting the River Otra, Norway [32], as well as in some hybrids between brown trout and brook trout (*S. trutta* x *Salvelinus fontinalis* [33]).

Morphological differences in chromatophore ultrastructure, and position or arrangement, or both, in the dermis of marble and brown trout have been revealed using transmission electron microscopy [34]. In zebrafish, position, interaction and presence or absence of different pigment cells provide the basis for different skin pigmentation and pattern. In these two trout species, however, the position or arrangement of the chromatophores in differently pigmented skin regions is much more complex, with a higher level of organisation of chromatophores observed in the skin of brown trout than that of marble trout. Meanwhile, a new pigment cell type, erythrophore type 2, and its ultrastructure in brown trout have been described for the first time in salmonids [34].

Recently, microarray analysis has been undertaken to compare gene expression profiles of whole skin samples of marble and brown trout, with dermis and epidermis, and all differently pigmented regions, considered as a single sample [35]. Four candidate genes for labyrinthine skin pattern have been described—*hdac1*, *vps18*, *dct* and *scg2a*—and it has been proposed that the formation of the labyrinthine pattern at least partially depends upon the Wnt signalling pathway and is based upon a reaction–diffusion mechanism. Only genes involved in the biological process ‘pigmentation during development’ were considered as informative [35]. In the present study, to further explore the pathways and genes expressed in the skin and potentially causative to skin pigment pattern in *Salmo*, we employed high-throughput mRNA sequencing technology on differentially pigmented dermis sections of marble trout and brown trout. These first RNA-seq data on differently pigmented dermis regions for these two species enabled (i) an overview of the transcriptome in dark, white and red coloured skin regions, and (ii) identification of differentially expressed genes (DEGs) possibly associated with skin pigment pattern. The expression profile of 27 DEGs was tested further with quantitative real-time PCR (qPCR) on a larger number of samples, while expression of a subset of ten of these genes was analysed in hybrid trout individuals (marble x brown trout) and compared with the complexity of their skin pigment pattern.

## Results

### Illumina sequencing, data assembly and annotation

Sequencing of RNA isolated from trout dermis resulted in a total of 812,144,586 pair-end reads of 100 bp each (414,612,488 in marble trout and 397,532,098 in brown trout) that were mapped to the gene model of Atlantic salmon (*S. salar*) genome (INSDC: AGKD00000000.4) [36]. All transcripts of the gene model were annotated with Swissprot to obtain the Gene symbols for Gene Ontology analysis. The assembled transcriptomes were submitted to the SRA database under accession number SRP157513. Results of transcriptome sequencing and mapping to the salmon genome are reported in Additional file 1.

### Genes highly expressed in trout skin

The top 50 genes most highly expressed in trout skin included genes of the keratin family and collagen proteins, with keratin, type II cytoskeletal cochlear-like the most highly expressed. The other genes highly expressed in marble and brown trout skin are reported in Additional files 2 and 3.

### Differential gene expression

Using NOISeq numerous DEGs between marble and brown trout pigment cell genes were identified. A total of 52,012 predicted genes were identified as expressed differentially between marble and brown trout skin. Of these, 3715 genes were up-regulated (≥ 2 fold) and 4327 down-regulated (≤ 2 fold) in the skin of marble trout compared to that of brown trout. Among the up- and down-regulated genes several are known to encode membrane proteins involved in, for example, signalling, cell junctions, or ion permeability. The 100 most up- and down-regulated genes that differed between marble and brown trout samples are reported in Additional files 4 and 5. Several key genes involved in skin pigmentation (e.g. *dct, tyr, tyrp1, pmel, ednrb*) showed differences in expression profiles between the two species, as did some genes involved in cell–cell junctions (e.g. *gja1, gja5, gjb6, gjd2, mpp3, tjp1*) and the establishment of membrane and action potentials (e.g. *clcn2*, *ctr2*, *kcnk3*). Among these groups of differentially expressed genes, 27 were selected for additional testing by qPCR.

K-mean clustering was used to group genes with a similar pattern of expression among samples. Many genes were overexpressed specifically in the red spot of brown trout compared to other skin regions of both species, indicating that there are specific pathways involved in either carotenoid metabolism or communication between these specific pigment cells and their surrounding cells, or both, resulting in the formation of a red spot.

### Functional classification of DEGs

DEGs were annotated through GO classification analysis and grouped into three categories (biological process, cellular component and molecular function) based on their putative functions. Salmon genes were annotated with BLAST [37, 38] against Swissprot and Swissprot Gene Ontology annotations at http://www.geneontology.org.

**Fig. 2:**
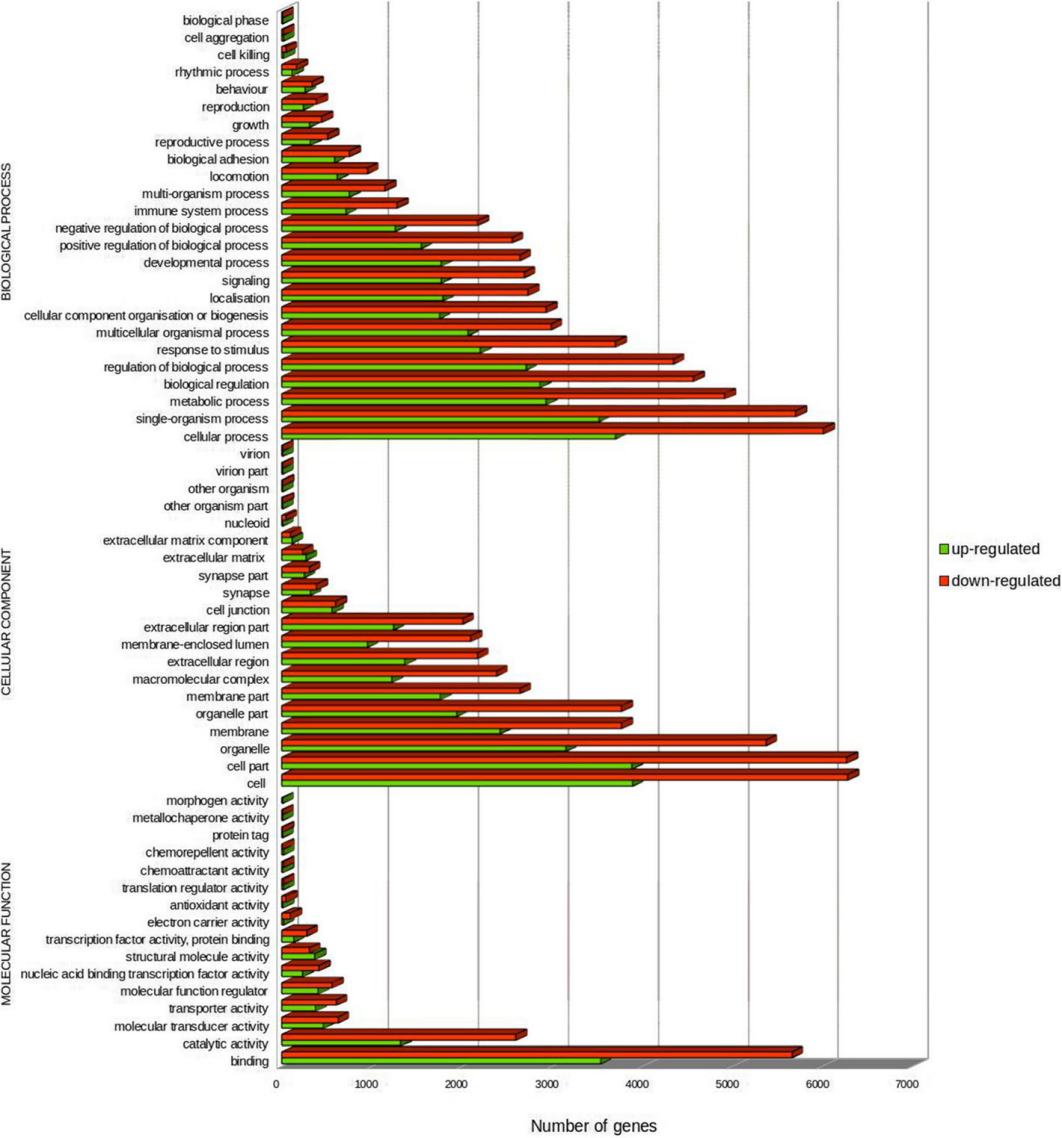
GO functional classification of transcripts according to their assigned molecular function, cellular component and biological process (up-regulated, MT > BT; down-regulated, BT > MT)

For the GO analysis, 18,087, 61,751 and 87,737 DEGs between marble and brown trout were grouped by molecular function, cellular component and biological process categories, respectively. Altogether, DEGs were mapped to 61 categories, as presented in Fig. 2. The GO terms containing the largest number of DEGs were cell, cell part, cellular process, single-organism process, binding and membrane.

### KEGG pathway analysis

**Fig. 3:**
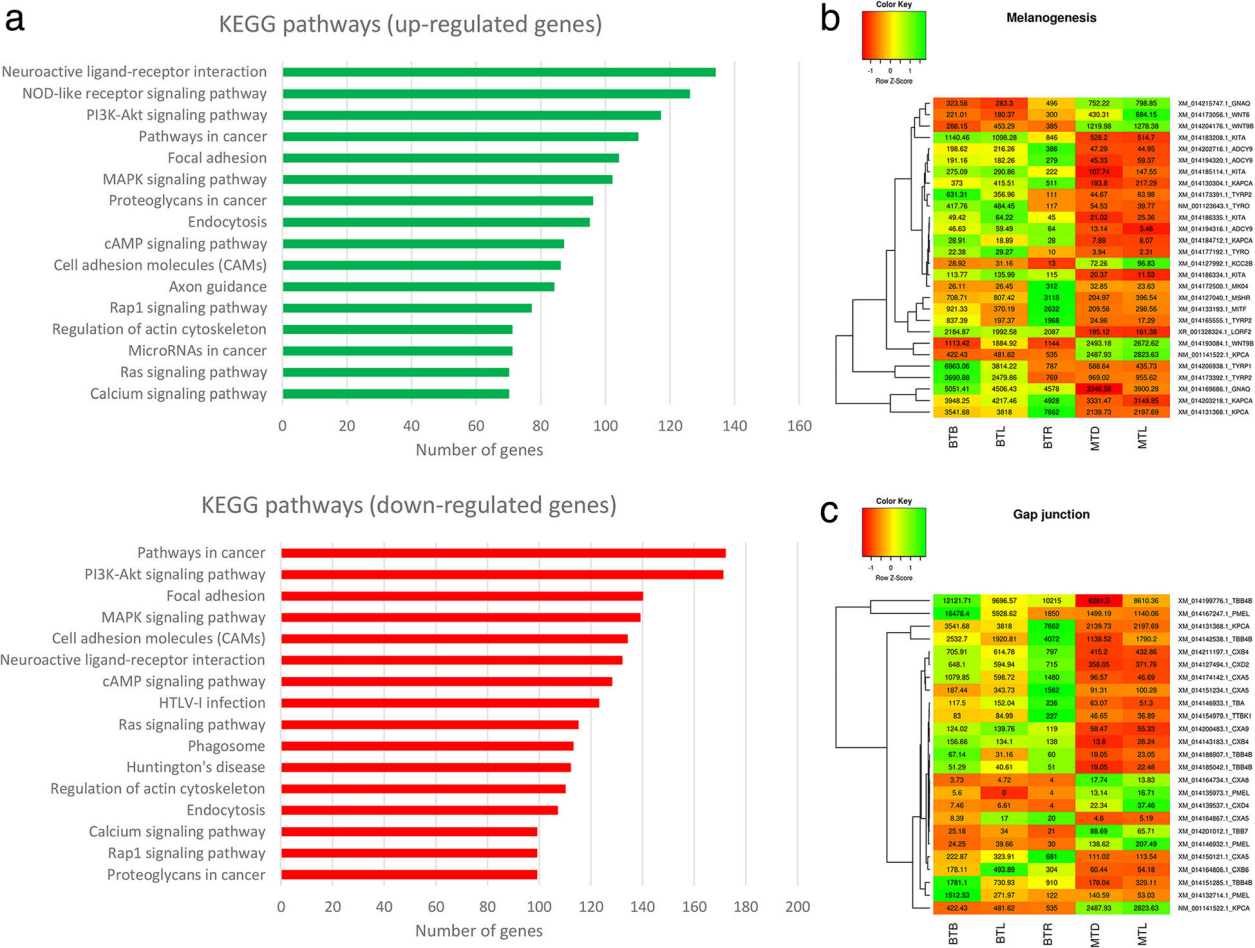
KEGG pathways: **a**, KEGG pathway classification of genes (green, up-regulated, MT > BT; red, down-regulated, BT > MT); **b**, Heat map visualisation of the expression of genes involved in melanogenesis; **c**, Heat map visualisation of the expression of genes involved in gap junctions. The columns display the samples and the rows the genes (red, down-regulated; green, up-regulated)

KEGG pathway analysis was carried out to categorise and annotate the DEGs, which were mapped and visualised in a way similar to the KEGG Mapper tool. The pathways with the largest number of genes expressed differentially between marble and brown trout were Neuroactive ligand-receptor interaction, NOD-like receptor signalling pathway, PI3K-Akt signalling pathway, Pathways in cancer, Focal adhesion, and MAPK signalling pathway (Fig. 3a). Other interesting differentially expressed pathways included: Cell adhesion molecules (CAMs), Calcium signalling pathway, Tight junction, Adherens junction, Gap junction, Cell cycle, p53 signalling pathway, Wnt signalling pathway, Melanogenesis and Tyrosine metabolism, all of which could affect and explain the difference observed between the two species in their skin pigment cell organisation. Expression profiles of DEGs from Melanogenesis and Gap junction are presented in Fig. 3b and c. The most represented pathways included, interestingly, the Thyroid hormone signalling pathway, Insulin signalling pathway and Insulin secretion. Many genes involved in these pathways were detected also during analysis of the first 100 up- and down-regulated transcripts with the highest fold change.

When comparing only the dark pigmented regions of the two species, the pathways with the largest number of DEGs were those describing neurodegenerative disorders, such as Huntington’s, Alzheimer’s and Parkinson’s diseases. When comparing only the light regions, the highest represented pathways were those associated with cancer, PI3K-Akt signalling, Focal adhesion, Huntington’s disease and Alzheimer’s disease.

The highest absolute number of DEGs was observed when comparing red skin with other skin regions in brown or marble trout. Focal adhesion, ECM receptor interaction, Cell adhesion molecules and MAPK signalling pathway were among the pathways with the largest number of genes represented and with a large differential expression profile.

### Quantitative real-time PCR

**Fig. 4:**
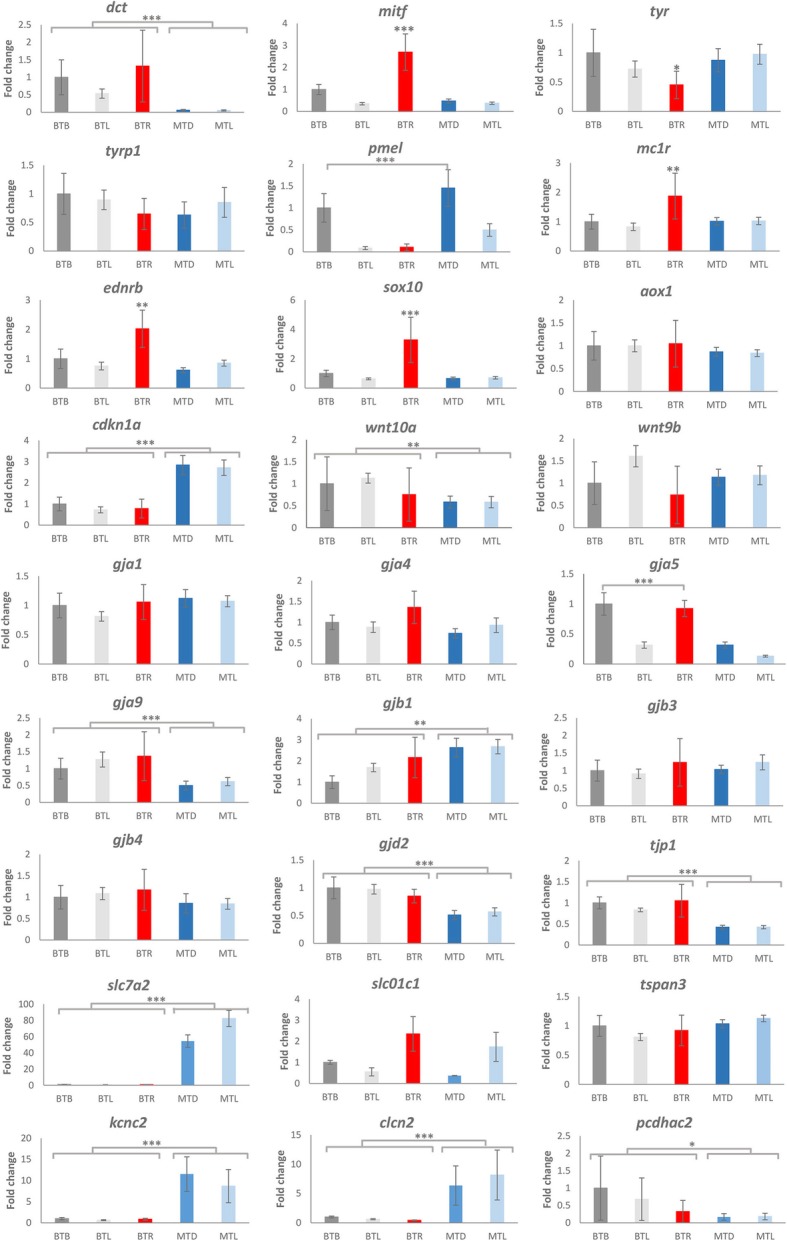
Gene expression patterns obtained with qPCR. Log2 fold changes are expressed as the ratio of gene expression after normalisation to reference genes. Bars represent the means ± SD. Grey, brown trout dark spot; light grey, brown trout light region; red, brown trout red spot; blue, marble trout dark region; light blue, marble trout light region. Expression of each gene was compared with expression in the black spot region of brown trout and statistically evaluated by using unpaired Student’s *t*-test. A *p*-value of < 0.05 was considered statistically significant (*p < 0.05; **p < 0.01; ***p < 0.001)

In order to validate the transcriptome sequencing results, we selected 27 DEGs for qPCR to determine their relative expression in marble and brown trout skin (Table 1). These genes could be classified into two groups based on their known functions in model organisms, including zebrafish: (1) genes involved in pigmentation pathways—*tyr*, *tyrp1*, *dct* (*tyrp2*), *mitf*, *pmel*, *mc1r*, *ednrb*, *sox10*, *aox1, cdkn1a, wnt10a* and *wnt9b*—, and (2) genes with a molecular function in cell binding known to be involved in ion exchange, chromatophore interactions and pigment pattern: *gja1*, *gja4*, *gja5*, *gja9*, *gjb1*, *gjb3*, gjb4, *gjd2*, *tjp1*, *tspan3, slc7a2, slc01c1, kcnc2, clcn2* and *pcdhac2*. qPCR (Fig. 4) showed that six (*dct*, *wnt10a*, *gja9*, *gjd2*, *tjp1, pcdhac2*) of the 27 selected genes had a significantly higher level of expression in brown trout skin than in marble trout skin, four others (*mitf*, *mc1r*, *sox10*, *ednrb*) were expressed significantly more, and one (*tyr*) significantly less, in the red spot of brown trout, while five genes (*cdkn1a, gjb1, slc7a2, kcnc2, clcn2*) had significantly higher expression levels in the skin of marble trout than in brown trout. Gene *gja5* exhibited an interesting pattern of expression, significantly higher in (black and red) spots of brown trout than in other skin parts of either species. The qPCR results were mostly consistent with the data from transcriptome sequencing, with 23 out of 27 genes having the same expression profile.

**Table 1:** Primers used for the qPCR analysis

Gene symbol	Gene accession no.	Gene description	Forward primer	Reverse primer	Efficiency (%)
*rps20*	NM_001140843.1	40S ribosomal protein S20	AGCCGCAACGTCAAGTCT	GTCTTGGTGGGCATACGG	99.8
*pgk*	XM_014138014.1	Phosphoglycerate kinase	CTCGGTGATGGGGCTTAGG	TCATTGGTGGAGGCGACA	98.1
*dct*	XM_014165555.1	L-dopachrome tautomerase-like	ACATGGCCTGATTCTACAGAGGCTCA	TCAGACCCGAGGGCAGGGC	110.3
*mitf*	XM_014133193.1	Microphthalmia-associated transcription factor-like	TGAGGACAGAAGGGGGTCAT	TGCGATTCTGCCATCGTCTT	93.5
*tyr*	NM_001123643.1	Tyrosinase	CAGCGGTTCATTGCCTACCT	GTCCGGCCAAACATTTCCTG	96.4
*tyrp1*	XM_014206938.1	Tyrosinase related protein 1b	TGGGAGAACAGTACCAACGC	TGCAAAAAGCGTGCCCTAAC	99.3
*pmel*	XM_014132714.1	Melanocyte protein PMEL-like	TTAAGCAGTATCGCCCACTGA	CTGTCGGCTCAGAAGATTCCA	93.5
*mc1r*	XM_014127040.1	Melanocortin 1 receptor	ATGCGATCCAGCACCAATGA	GAAGAGCCCAGATGCTCACA	96.5
*ednrb*	XM_014191488.1	Endothelin B receptor-like	AGCAATTTAGCGAAGGCGTG	CTGTGGCAGAGGCGTCATAG	109.7
*sox10*	XM_014203766.1	Transcription factor Sox-10-like	TAGCTGGCGGGATTTCCATC	GAGGTGCGGATACTGGTCTG	99.7
*aox1*	XM_014151595.1	Aldehyde oxidase 1-like	AGTGACCAACATGCTCTGGG	ACGCCAACTCTGGAAACACT	116.2
*cdkn1a*	XM_014166886.1	Cyclin dependent kinase Inhibitor 1-like	TGGGGCTTTGATTTCCTGTCA	AAAAGGCAGGGTTGGTAGAGG	106.4
*wnt10a*	XM_014164238.1	Protein Wnt-10a	GACCCCATTCTCAACGCCAA	TATGCAAAAGCACTCTCACGG	117.5
*wnt9b*	XM_014204176.1	Protein Wnt-9b-like	GCCAGAAGAGGGTCAGCAAA	TCGTAGCGGAACTTGAGCAG	100.2
*gja1*	XM_014144390.1	Gap junction alpha-1 protein	TAGTTGCGAACGGAGTTGGT	TGGCACTGGGACATTAAGCC	87.4
*gja4*	XM_014178807.1	Gap junction alpha-4 protein-like	ATAGCCGTGGTTCCAATCCG	GAATGTCACCACCAGGGTCC	102.5
*gja5*	XM_014174142.1	Gap junction alpha-5 protein-like	TGCCTTCTCACCCCTTCAAC	CCGGTATGCAGTAAGGGTGG	91.2
*gja9*	XM_014200483.1	Gap junction alpha-9 protein-like	CGATGCCCGTGTAAAGATGG	CGTGTTGCAGATGAAGTCGG	112.4
*gjb1*	XM_014137501.1	Gap junction beta-1 protein-like	CTGCGATGTTGAGCAGGATG	AAGTGTGACTCGTACCCCTG	101.5
*gjb3*	XM_014157241.1	Gap junction beta-3 protein-like	TGGTACTTCCGCTCACGTTC	AAGTACTCCACTGCGTTCGG	96.7
*gjb4*	XM_014143183.1	Gap junction beta-4 protein-like	TTGCGTGAGGGGAAGGATTC	GACGGGAGATGTAGCAGTCG	99.7
*gjd2*	XM_014127494.1	Gap junction delta-2 protein-like	TTGCACCGGTCGAATCC	AGAGTGTACGGGTAGGGACT	117.5
*tjp1*	XM_014170256.1	Tight junction protein 1	TATGGCCTTGGTTCGCTGTA	GGTCAGTTCCAAGGCTCACG	109.8
*tspan3*	XM_014148589.1	Tetraspanin 3	ACACCCTTATCCCTGCTGTG	CCGTAGCGGCTTTCTCGTAT	99.6
*slc7a2*	XM_014198602.1	Cationic amino acid transporter 2-like	GTGTGCTTGCTTCAGCTATCC	GTGCTTGCTGACCAAGTTGT	89.0
*slco1c1*	XM_014186199.1	Solute carrier organic anion transporter family member 1C1-like	AACTTCTTCTGCCGCTTGGA	ACGTTCCTGGGTCTGGTCTA	92.3
*kcnc2*	XM_014173424.1	Potassium voltage-gated channel subfamily C member 2-like	TCCTGACGACCTTTAGGGCA	TGTTGCAGTGGTGTAGCCTT	94.8
*clcn2*	XM_014190851.1	Chloride channel protein 2-like	TTGCTGTATGTTCTCGGGCG	TGGACCTGCCATCGGAATTG	92.9
*pcdhac2*	XM_014212430.1	Protocadherin gamma-C3-like	GGAAGGCTCCGTTGTTGCTA	AGCAAGTGGTCGTTTTGATG	106.4

### Correlation between expression of candidate genes and phenotype of hybrids

From analysis of the qPCR products, ten candidate genes (*dct*, *cdkn1a*, *gja5*, *tjp1*, *wnt10a*, *gjd2, slc7a2, kcnc2*, *clcn2* and *pcdhac2*) with the most differential expression between the two species were selected and their expression further tested in differently pigmented skin regions of hybrid samples.

**Fig. 5:**
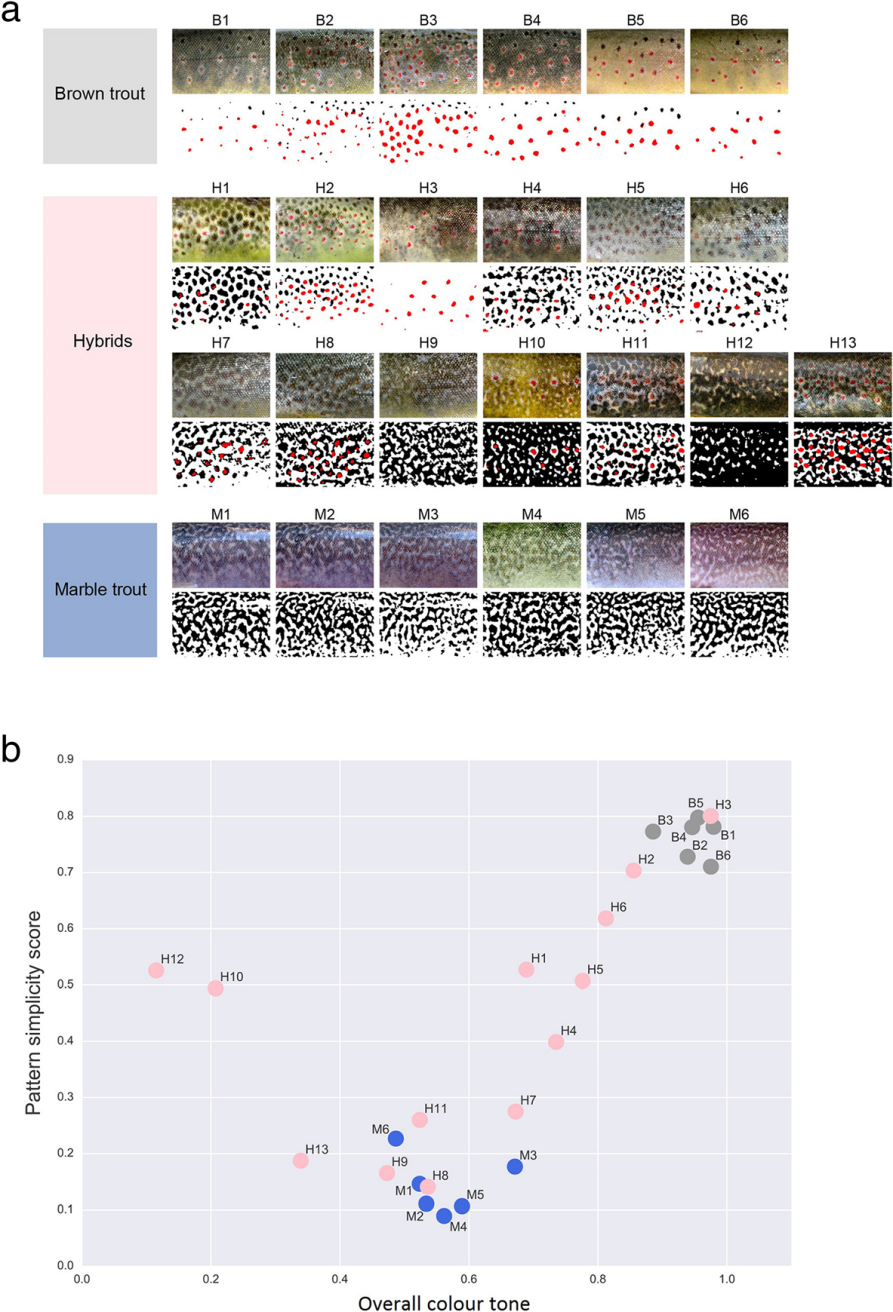
Skin pigment pattern variation: **a**, Skin pigment pattern variation in genetically pure marble and brown trout, and in hybrid animals; **b**, quantification of pigment patterns in hybrids (pink dots) displaying different patterns, ranging from brown trout phenotype (grey dots) to marble trout phenotype (blue dots)

**Fig. 6:**
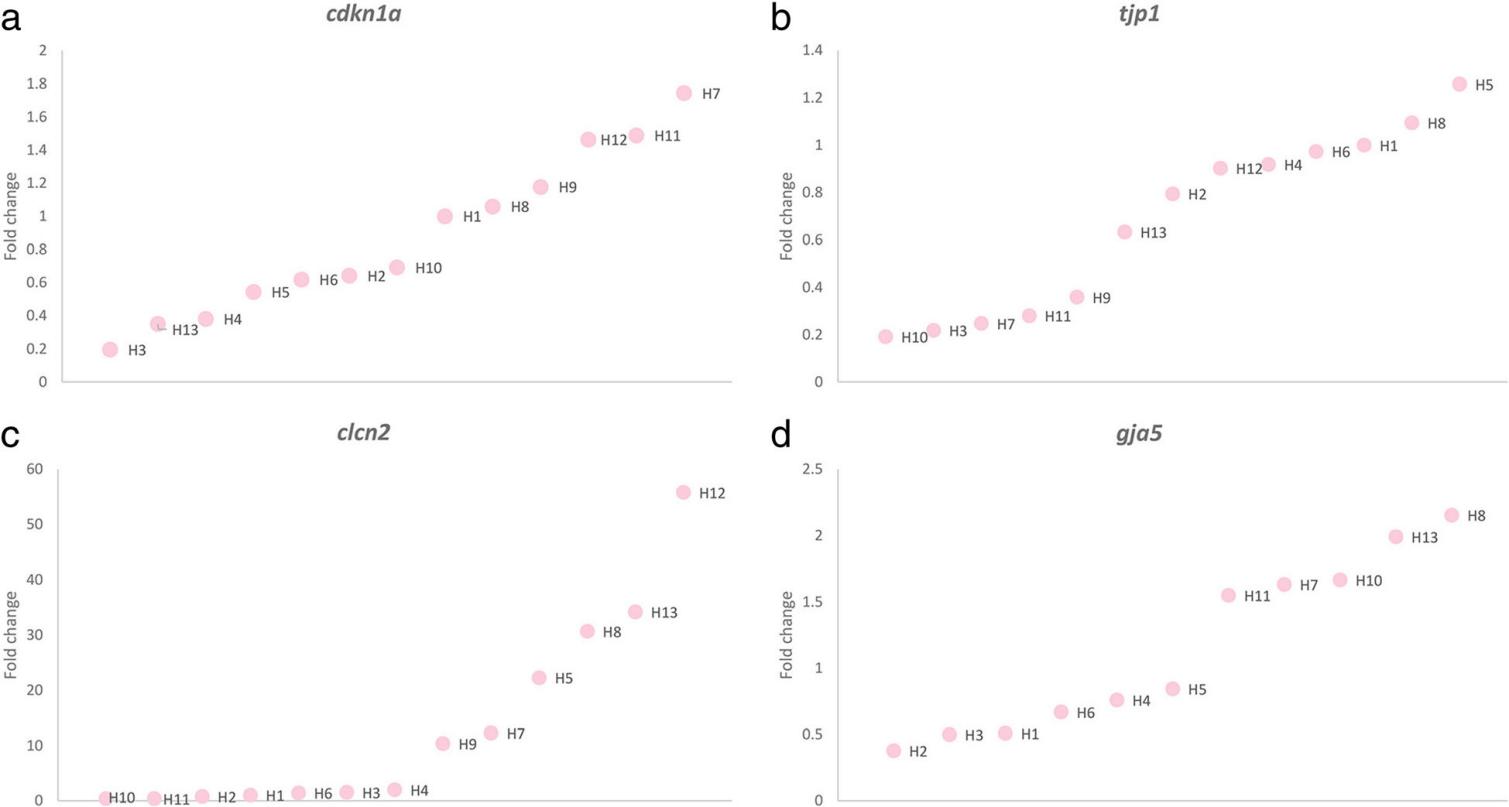
Gene expression patterns of four candidate genes in hybrids: **a**, expression of *cdkn1a* gene averaged over skin regions in hybrids; **b**, expression of *tjp1* gene averaged over skin regions in hybrids; **c**, expression of *clcn2* gene averaged over skin regions in hybrids; **d**, expression of *gja5* gene in the red spots of hybrids

The skin pigment pattern variation of hybrid animals was evaluated and compared to those of brown trout and marble trout. The complexity of colour pattern in each individual fish, with a lower score representing greater complexity, was plotted against the overall colour tone of the pattern, defined as the proportion of white area [33] (Fig. 5). The complexity of pattern variation of hybrids was then compared with the expression of candidate genes. The expression of *clcn2*, *cdkn1a* and *tjp1* was partially consistent with pattern complexity, while *clcn2* and *cdkn1a* demonstrated a higher level of expression in hybrids closer to the marble phenotype than to the brown trout phenotype, and *tjp1* had a higher expression in hybrids closer to the brown trout phenotype (Fig. 6). The overall expression of *gja5* was partly correlated with pattern complexity, though the red spot samples showed a strict overexpression in hybrids with a phenotype closer to marble trout (Fig. 6). Figures 5 and 6 show the average expression value for each hybrid, and expression values for every differently pigmented region of their skin is shown in Additional file 6. For other genes no correlation between expression profile and pattern complexity was observed.

## Discussion

Various mechanisms are thought to be responsible for the great variety in skin pigment patterns observed in animals. Recently, the role of homotypic and heterotypic intercellular interactions has been demonstrated in pigment progenitor cell migration, and in the formation and maintenance of a specific pigment pattern in zebrafish [10, 17–19]. In order to screen for genes potentially involved in maintenance of a specific pigment pattern in marble and brown trout, we conducted comparative transcriptome analysis of differently pigmented regions of the adult skin of the two species. The results indicate that the maintenance of different skin pigment patterns in trout is dependent upon specific communication—involving gap junctions, tight junctions and ion channels—between chromatophores present in differentially pigmented skin regions. Some candidate genes are involved in the distribution and differentiation of pigment progenitor cells [3, 39].

### DEGs and genes potentially correlated with skin pigment pattern

KEGG pathway analysis showed that a substantial number of DEGs between the two species are associated with pigmentation-related pathways—e.g. MAPK signalling pathway, Wnt signalling pathway, Melanogenesis, Tyrosine metabolism—and cell–cell communication, such as Focal adhesion, Cell adhesion molecules (CAMs), Calcium signalling pathway, Tight junction, Adherens junction and Gap junctions.

Differential expressions were also observed when differently coloured regions of skin from the same individual fish were compared, most probably associated with specific expression profiles of individual pigment cell types predominating in a particularly coloured skin region [34]. When comparing only dark regions or only light regions between the two species, several DEGs were found. These genes are involved in Ion binding and exchange, Ion flow, Cell–cell junctions, Cellular signal transductions (Ras subfamily of proteins) and Actin regulation (Rho GTPases). Genes involved in Melanogenesis (*pmel*, *tyrp1*, *dct*) were overexpressed in the black spot of brown trout when compared to other pigmented regions of brown trout skin.

The results from the present study indicate that DEGs with a possible role in trout skin pigment pattern maintenance can be classified into two major groups: (1) genes involved in pathways that have a known role in pigmentation (e.g. *mc1r*, *tyrp1, dct, wnt6*), and (2) genes with a molecular function of binding, including those coding for proteins involved in cell–cell communication and ion channels (e.g. *gjd*2, *clcn2*, *kcnk3*, *kcj12*), known to act as factors that can affect the organization of pigment cells in the dermis [3, 23]. We selected 27 genes from both groups for qPCR confirmation of differential expression. RNA isolated from differently pigmented regions of ten brown and ten marble trout samples were used for individual qPCR validation of the sequencing results. The expression profile of most of the DEGs were in agreement with the results of the comparative transcriptome analysis, indicating that there was no consistent bias in the expression profile.

In addition, expression of ten candidate genes, with consistent differences in expression between marble and brown trout skin, was analysed in hybrid individuals and correlation with phenotype complexity was assessed. The phenotype complexity was comparable with various levels of brown trout introgression (Additional file 7), estimated from the genotyping of 36 SNPs.

Of four differentially expressed informative genes—*hdac1*, *vps18*, *dct* and *scg2a*—previously proposed to be involved in skin pigment pattern maintenance in marble and brown trout [35], only *dct* demonstrated a large difference in the present study, and was expressed more than twice as much in the marble trout skin than in the brown trout skin. As explained below, different expression of this gene does not correlate with the phenotype of hybrid individuals, nor do the remaining genes that are involved in pathways that have a known role in pigmentation. These other genes are important for pigment synthesis; their differential expression thus results in different pigmentation intensity and, most probably, not in a different pigment pattern.

### Candidate genes determined from correlation between gene expression and phenotype

A positive correlation between phenotype and the expression profiles assessed in hybrid individuals with a variety of different skin pigment patterns was observed for four of the tested candidate genes: *gja5*, *clcn2*, *cdkn1a* and *tjp1*. Each of these genes is included in the above-mentioned second group of candidate genes, with a molecular function of binding and cell–cell communication. (However, their expression was not entirely consistent with the assessed complexity—or simplicity—of the pattern, perhaps from an accumulated bias from the steps of pattern evaluation: e.g. quality of photographs taken in the field; the dark parts of the hybrid skin not being separated into spotted and non-spotted regions; parr marks present in some hybrids affecting evaluation of the pattern.)

Gene *cdkn1a* (also referred to in the literature as *p21*) encodes a cyclin dependent kinase inhibitor 1a that was up-regulated in skin with labyrinthine pattern (marble trout). This gene functions as a regulator of cell-cycle progression in the phases G1 and S. Furthermore, it has been shown that *cdkn1a* is involved in patterning of eye-spots in the pigmented epithelium of the retina [40]. Another interesting role of this gene involves the regulation of cell migration and actin stress fibre formation through regulation of the Rho pathway [41], which could have a role also in skin pigment patterning. Finally, *cdkn1a* is known to be important in the development of melanocytes, promoting their differentiation and melanogenesis [42].

Šestáková et al. [43] reported a higher level of P21 protein in lightly pigmented, faster proliferating melanocytes than in dark-pigmented, slowly proliferating melanocytes. In a previous study, we observed a different ultrastructure of the melanophores present in the dark spot than in the light region of brown trout skin [34]. In addition, we noticed two types of melanophores—stagnate and active—present in the primary culture of pigment cells isolated from brown trout skin, with a slightly different colour and different speed of movement (data not shown, available from authors upon request). The presence of only one type of melanophore was noticed in the skin of marble trout, reflecting mostly the characteristics of the active type of melanophore. We therefore suggest that there may be up to two types of melanophore present in trout skin, with a higher share of slow, almost stagnate, melanophores in brown trout compared to a higher share of fast active melanophores in marble trout. A large proportion of the stagnate type melanophore in dark spots of brown trout skin indicates it could potentially be involved in spot maintenance. Also, the movement of the two types of melanophore may be differentially regulated through *cdkn1a* and the Rho-pathway and thus could affect the final skin pigment pattern. To substantiate our hypothesis KEGG analysis of the light region versus the black spot was conducted. Pathways with the largest numbers of DEGs included PI3K-Akt signalling pathway, Focal adhesion, ECM-receptor interaction, Cell adhesion molecules, Regulation of actin cytoskeleton and Axon guidance. These results show that different cell movement and attachments could indeed affect the perpetuation of spots.

A second candidate gene, *clcn2,* encodes a plasma membrane voltage-gated chloride channel Clcn2 (also known as ClC-2). As with other chloride channels, this is a transmembrane protein involved in the transport of chloride ions and, through that, the maintenance of chloride ion homeostasis and the transmission of electrical signals between cells. The functions of chloride channels include membrane potential stabilization, signal transduction, regulation of cell volume and of a tight junction, and transepithelial transport [44]. To the best of our knowledge, the *clcn2* gene has not been described previously to be involved in pigment patterning. Meanwhile, another chloride channel—CIC-7—has been shown to have an important role in pigmentation in mice [45], while in zebrafish a potassium channel—Kir7.1—has been connected to specific pigment cell interactions and depolarizations, affecting pigment patterning [20].

The other two candidate genes, *tjp1* and *gja5*, are involved in cell–cell junctions. The gene *tjp1* (tight junction protein 1, also known as *zo-1*) encodes a protein involved in tight junctions and was up-regulated in spotted skin (red and black spots in brown trout). It has already been demonstrated that the expression of this gene can affect pigment patterning. In a previous study, a zebrafish with a mutation that disrupts the functioning of the *tjp1a* protein was examined [21]. The mutant had a spotted pattern rather than the usual striped pattern. It was shown that *tjp1a* was expressed in dense iridophores but its expression was down-regulated in loose-form iridophores and in melanophores and xanthophores. In the mutant, the dense iridophores interrupted the dark stripes, which reorganized to form spots. Iridophores in trout skin were not recognized as loose or dense forms, but rather were classified based upon the size of their inclusions, into large (L) and small (S) types [34]. Nevertheless, we hypothesize that *tjp1* might play a distinct role in the functioning of these iridophores. Namely, type L iridophores were present exclusively in the light area of brown trout skin [34]. With the overexpression of *tjp1*, these cells could prevent melanophores from forming or from maintaining the labyrinthine pattern, or both, but rather organizing into spots. Another, very likely, role of the *tjp1* gene in skin pigment patterning is connected to the observation that Tjp1 protein is a scaffolding protein that links tight junction proteins (such as claudins and occludins) to the actin cytoskeleton and could, as a consequence, affect the rearrangement of the cytoskeleton and the movement of the cells [46]. Together with *cdkn1a*, which has a similar role, the expression profile of these two genes could explain the diverse movements of two types of melanophores and their communication with other pigment cells resulting in different pigment patterns [47, 48].

The gene *gja5* (gap junction protein alpha 5, also known as *cx40*) was overexpressed in dark and red spots in brown trout. This gene codes for a protein that is a member of the connexin family, which forms another type of cell junction: gap junctions. These are connections between cells, have a role in cell–cell communication and are involved in the maintenance of ionic and metabolic homeostasis and transmission of various ions and electrical signals [49]. Dark and red spots in brown trout are characterized by both melanophores (stagnate, see above) and erythrophores [34]. Thus, it is hypothesized that *gja5* might play an important role in communication (heterotypic interaction) between these two cell types or between erythrophores (homotypic interaction), resulting in a spotted pattern. The role of gap junction proteins in pigment pattern formation and maintenance has been confirmed several times in zebrafish, mainly through studying a range of mutants with various pigment patterns as a consequence of the modified *cx41.8* (also known as *gja5b*) gene (missense point mutations, deletions) [3, 22, 23, 50], a homolog of *gja5*. Nevertheless, to the best of our knowledge, this is the first study demonstrating a connection between *gja5* expression level and skin pigment pattern. Differential expression of another connexin—*gja1*—between light and dark pigmented regions has been shown previously in sticklebacks [51], where a higher level of expression was detected in tissue from light bars. The high expression level of *gja5* in red spots of hybrids showed a direct correlation with phenotype, with an overexpression in red spots present in skin with a general marble trout phenotype, or labyrinthine pattern. We additionally examined the correlation between *gja5* expression in red spots and overall colour tone that is directly connected to the labyrinthine pattern. The hybrids formed two groups, where higher *gja5* expression was connected to a darker overall colour tone (Additional file 8). A possible explanation for this, at first sight paradoxical, result involves cell interactions and the specific cell environment [52–54], with possible indirect mechanisms involved in pigment pattern maintenance and chromatophore interactions [13–16, 18]. Erythrophores forming red spots in brown trout and some hybrid individuals are otherwise not present in marble trout skin (labyrinthine pattern). We hypothesise that more intense specific homotypic (between erythrophores) or heterotypic (erythrophore–melanophore), or both, interaction and molecular or signal transductions are required in hybrids in order to maintain the red spot pattern on a labyrinthine pattern background. Since the gap junction works as a channel, connecting the cytoplasm of two cells and permitting the passage of various ions and molecules, and is affected by depolarisation, the roles of all four candidate genes for pigment pattern maintenance in *Salmo* could be tightly entwined. It has been shown that melanophores present in zebrafish jaguar mutants, with a mutation in the potassium channel Kir7.1, were constantly depolarised, and not only when in contact with xanthophores, as observed in wild-type melanophores [55]. The consequence of this constant depolarisation is a variant pattern in jaguar mutant skin, where usual zebrafish stripes are interrupted. On the other hand, depolarisation could also affect the actin cytoskeleton rearrangement [56]. The involvement of all four candidate genes suggests the importance of bioelectrical signalling, depolarisation, cytoskeleton rearrangement and cellular movement in skin pigment pattern maintenance in trout. To confirm this hypothesis further studies need to be conducted.

### Conclusion

In summary, we sequenced transcripts from the skin of marble trout and brown trout for the first time and identified more than 8000 DEGs (with a fold change ≥2) in the skin of the two species. These DEG data provide a reference for screening genes for their effect on pigment patterning and other processes taking place in trout skin. The results significantly enhance our understanding of the composition of the trout skin transcriptome and the potential differences in gene expression associated with skin pigment pattern maintenance, and provide a foundation for future studies. We proved the validity of the mRNA-seq results using qPCR, where 27 candidate genes were tested on a large number of samples. Our results suggest an important role of cell–cell interactions, junctions and cytoskeleton rearrangements in trout skin pigment pattern maintenance as a consequence of differential gene expression.

## Methods

### Experimental animals

Adult marble trout individuals (of age > 2 years) were collected from Tolminka fish farm in Tolmin, Slovenia, and adult brown trout (of age > 2 years) from Bled fish farm, Bled, Slovenia. With an age of at least two years these trout had reached sexual maturity and already developed their adult skin pigment pattern. Their body length was approximately 30 cm. All fish were first-generation offspring reared in their respective fish farm from wild-caught parents originating from Zadlaščica stream (marble trout) and Malešnica stream (brown trout), Slovenia. Individuals had been fed with Biomar INICIO Plus (Denmark) as a starter feed and Biomar EFICO Enviro 920 when adult. Prior to skin sample collection fish were sedated in anaesthetic Tricaine-S (MS-222, Western chemical, Ferndale, USA) and killed by a blow to the neck.

In order to verify any correlation between the expression of ten candidate genes and the skin pigment pattern, hybrids between the two species with a variety of pigment patterns were sampled from Volarja stream (Soča river basin, Slovenia) where stocking with brown trout has been intensive for several decades and where ‘pure’ marble trout could not be found due to hybridisation. Hybrids with various levels of brown trout introgression are a result of hybridisation between marble and introduced brown trout, and backcrosses. Level of introgression was assessed by genotyping a panel of 36 diagnostic nuclear SNPs as reported previously [57]. The introgression levels for hybrid individuals are reported in Additional file 7. Skin from thirteen hybrid trout was selectively dissected with biopsy punches from differentially pigmented parts (light and dark region, red spots where present). In total, 37 different samples were collected.

All methods described were carried out in accordance with relevant guidelines and regulations. Fish skin sampling was approved by the Ministry of Agriculture and Environment, Slovenia, under decision letter number U34401–60/2013/4.

### Sample preparation

Small pieces of skin from the lateral part of the trunk of the body (Fig. 1) were obtained with a 2 mm biopsy punch (Kai Group, Japan) to selectively dissect differently pigmented regions of skin in marble trout (dark (MTD) and light (MTL) region) and brown trout (light region (BTL), black (BTB) and red (BTR) spots). Skin pieces were immediately immersed in liquid nitrogen and subsequently used for preparation of cryosections. Only sections of the dermis where pigment cells are located (see [34] for more details about pigment cell location) were used for total RNA extraction (Fig. 1).

### Total RNA extraction and transcriptome sequencing

Total RNA was isolated from cryosections of skin using a PicoPure RNA Isolation Kit (Thermo Fisher Scientific, Waltham, MA USA) according to manufacturer’s instructions. All samples were treated with DNA-free DNA Removal Kit (Thermo Fisher Scientific) to remove traces of genomic DNA. Concentration of total RNA and RNA integrity value (RIN) were checked using an RNA 6000 Pico LabChip of Agilent 2100 Bioanalyzer (Agilent Technologies, Santa Clara, CA, USA). RNA extracts with the highest RNA integrity of identically pigmented skin samples from three individuals per species were pooled. All RIN values were > 8.5. In total, five libraries from 1 μg of total RNA isolated and pooled from six individual fish (three per species), labelled as MTD, MTL, BTB, BTL and BTR, were prepared using random hexamer primers. cDNA libraries were sequenced by Beijing Genomics Institute (Hong Kong, China) using 2x100bp pair-end high-throughput mRNA sequencing (RNA-seq) on two lanes (1st lane with two marble trout samples, 2nd lane with three brown trout samples) of Illumina Hiseq 2000 (Illumina, San Diego, CA).

### Bioinformatical analysis of RNA-seq

Paired end sequences from the mRNA-seq were mapped with Bowtie2 [58] against the transcripts (gene model) of Atlantic salmon genome (ICSASG_v2) applying standard parameters, and a raw count table was extracted using SAMtools and the command ‘Samtools idxstats’ (SAMtools version 0.1.19-96b5f2294a). Reads were mapped directly to the gene model of Atlantic salmon, as this proved to be less error prone than mapping to the genome due to sequence differences between Atlantic salmon and marble and brown trout and difficult prediction of exon/intron boundaries. The read counts were normalized to total reads in the sample with the smallest number of reads. Genes for which the read count was less than ten in the highest expressed sample were discarded and differentially expressed genes (DEG) were computed with NOISeq [59].

### Differential gene expression

Differential expression analysis was performed using the R/Bioconductor package NOISeq (1-PNOI < 0.05, equivalent to false discovery rate [FDR] adjusted *P*-value [60, 61]). For NOISeq, the probability of a gene being differentially expressed that was provided by the method was used for the threshold (0.8). Since there were no true biological replicates available, technical replicates were simulated by NOISeq-sim, where single samples were used in NOISeq and additional samples from the same species run as replicates in making comparisons between the species. As NOISes-sim only simulates technical replicates, we accounted for some of the biological variability by using RNA from three individual fish per sample for transcriptome sequencing. The differential expression of selected genes was validated by running qPCR on multiple samples. qPCR also provided information on the biological variation of the genes of interest. We ran NOISeq for comparison on pooled libraries, using the library-normalised raw read count for each gene. K-mean clustering was used to group genes with similar patterns of expression. Gene Ontology [62, 63] was used to pick out genes that were annotated with ECM. Gene model transcripts of Atlantic salmon were annotated by BLAST+ against Swissprot to obtain the gene symbol for each reference gene. Gene Ontology annotation terms for Swissprot were downloaded from www.geneontology.org. The output ECM gene list could be cross-checked for DEGs from NOISeq. KEGG pathway analysis [64] was performed by mapping the KEGG annotated DEGs from NOISeq to KEGG pathways as described in the KEGG Mapper tool. Both raw expressions of genes and DEGs as fold change were plotted in pathways, and the ratio of number of up-regulated genes to number of down-regulated genes, or vice versa, were calculated as a means of ranking up- or down-regulated pathways.

### Quantitative real-time PCR

Samples for RNA isolation were prepared using biopsy punches to selectively dissect differently pigmented parts of the skin. Skin pieces were then homogenised with Percellys24 homogenizer (Bertin corp.) with the use of zirconium oxide beads (Bertin corp). Total RNA was isolated using RNeasy Plus Universal Mini Kit according to manufacturer’s instructions and subsequently treated with RNase-free DNase I (Thermo Fischer Scientific). Only RNA with good purity, checked with a NanoVue spectrophotometer (GE Healthcare, Little Chalfont, Buckinghamshire, UK), with values A260/280 ~ 2.0 was used for cDNA synthesis, performed with High-Capacity cDNA Reverse Transcription Kit (Thermo Fischer Scientific).

qPCR was performed using SYBR Green PCR Master Mix (Thermo Fischer Scientific, MA USA) and conducted on a Viia7 Real-Time PCR System (Applied Biosystem, Thermo Fisher Scientific). All primers for selected candidate genes (Table 1) were designed based on transcriptome sequences (SRP157513) obtained using Primer-BLAST [65] with an amplicon size of approximately 100–200 bp. Ten potential reference genes—*rps20*, *hprt1*, *RNA polII*, *ppia1*, *pgk*, *sdha*, *b2m*, *ef1a*, *β-actin*, *18 s*—were analysed to identify two that could be used as reference genes: *rps20* and *pgk1* were chosen as having a stable expression, i.e. at the same level in all tested samples. The expression of candidate genes was analysed with ten biological replicates of marble (dark, MTD; light region, MTL) and ten of brown trout (light region, BTL; black spot, BTB; red spot, BTR), each performed in triplicate. A no-template-control (NTC) was used to check for potential external contamination. Standard curve analysis was performed for reference and target genes to assess amplification efficiency, which was comparable between genes. The conditions for all reactions were 50 °C for 2 min, 95 °C for 10 min, followed by 40 cycles of 95 °C for 15 s and 60 °C for 1 min. At the end of every run, melting curve analysis was performed in order to confirm a unique amplicon reaction.

Expression of ten candidate genes with the highest differential expression between the two species was additionally tested in differently pigmented skin regions of hybrid samples using the protocol described above.

Differential gene expression results were calculated using the Pfaffl method [66], normalised to the geometric mean of the above-mentioned reference genes and with the use of amplicon-specific efficiency. Student’s t-test was performed to assess the statistical significance of differential expression between samples, and results with *p* < 0.05 were considered statistically significant.

### Quantification of pigment patterns

Pigment patterns of hybrid animals and genetically pure brown and marble trout were quantified and analysed as described previously [33]. Briefly, colour pattern complexity, overall tone and the proportion of red spot regions were quantified from images using in-house Python scripts and the OpenCV library. Pattern simplicity score (*PSS*) is defined as the area-weighted mean isoperimetric quotient of the contours extracted from each image:

$$ PSS=\sum \limits_i{w}_i\ {Q}_i, $$

where ${Q}_i=\frac{4\uppi {S}_i}{L_i^2}$ is the isoperimetric quotient (or circularity) of each contour, ${w}_i=\frac{S_i}{\sum \limits_i{S}_i}$ is the area weight, and *S*_*i*_ and *L*_*i*_ are, respectively, the surface area and the perimeter of each contour. The overall colour tone of a pattern was defined and calculated as the proportion of non-pigmented area (white pixels in a binarized image). The proportion of red spot regions was calculated from the average proportion of reddish pixels in each image.

## Additional files

Additional file 1: **Table S1.** Summary results of transcriptome sequencing and mapping. (PDF 21 kb)

Additional file 2: **Table S2.** 50 most expressed transcripts in marble trout. (PDF 51 kb)

Additional file 3: **Table S3.** 50 most expressed transcripts in brown trout. (PDF 61 kb)

Additional file 4: **Table S4.** 100 transcripts with the highest fold change between marble and brown trout samples. (PDF 108 kb)

Additional file 5: **Table S5.** 100 transcripts with the highest fold change between brown and marble trout samples. (PDF 111 kb)

Additional file 6: **Figure S6.** Gene expression patterns of four candidate genes in differently pigmented regions in the skin of hybrids. Red, red spot; dark brown, dark region; light brown, light region. (PDF 345 kb)

Additional file 7: **Table S7.**. Level of brown trout introgression in hybrid individuals. (PDF 417 kb)

Additional file 8: **Figure S8.** Correlation between the expression of *gja5* gene in the red spots of hybrids and overall colour tone of the skin in hybrids. (PDF 315 kb)

## Abbreviations

BTB: brown trout black spot skin region
BTL: brown trout light skin region
BTR: brown trout red spot skin region
CAMs: Cell adhesion molecules
cDNA: complementary DNA
DEGs: differentially expressed genes
DNA: Deoxyribonucleic acid
GO: gene ontology
KEGG: Kyoto Encyclopedia of Genes and Genomes
mRNA: messenger ribonucleic acid
MTD: marble trout dark skin region
MTL: marble trout light skin region
NTC: no-template-control
PCR: polymerase chain reaction
PSS: Pattern simplicity score
qPCR: quantitative real-time PCR
RIN: RNA integrity value
RNA: ribonucleic acid
RNA-seq: ribonucleic acid sequencing
SNPs: single nucleotid polymorphisms
type L iridophores: large iridophores
type S iridophores: small iridophores
WGD: whole genome duplication
