# Supplementary material for: Comparative transcriptome analysis of trout skin pigment cells
Source: BMC Genomics. 2019 May 9;20:359. doi: 10.1186/s12864-019-5714-1 (PMC6509846; doi:10.1186/s12864-019-5714-1)
Supplement: Supplementary file 1 — Table S1. Summary results of transcriptome sequencing and mapping. (PDF 21 kb) [file 12864_2019_5714_MOESM1_ESM.pdf]

Table S1

Summary results of transcriptome sequencing and mapping.

	Total reads	*Mapped reads*	*Mapped %*
Marble trout skin, dark region	193864706	153033795	78.94%
Marble trout skin, light region	220747782	174433454	79.02%
Brown trout skin, black spot	136690754	107814100	78.87%
Brown trout skin, light region	134345744	106462637	79.25%
Brown trout skin, red spot	126495600	100537845	79.48%
