# Supplementary material for: Comparative transcriptome analysis of trout skin pigment cells
Source: BMC Genomics. 2019 May 9;20:359. doi: 10.1186/s12864-019-5714-1 (PMC6509846; doi:10.1186/s12864-019-5714-1)
Supplement: Supplementary file 2 — Table S2. 50 most expressed transcripts in marble trout. (PDF 51 kb) [file 12864_2019_5714_MOESM2_ESM.pdf]

Table S2

50 most expressed transcripts in marble trout.

Transcript	Annotation	Genesymbol
XM_014147199.1	PREDICTED: Salmo salar keratin, type II cytoskeletal cochleal-like (LOC106572750), mRNA	KRT8
XM_014135683.1	PREDICTED: Salmo salar keratin, type II cytoskeletal cochleal-like (LOC106566974), mRNA	KRT8
XM_014176408.1	PREDICTED: Salmo salar keratin, type I cytoskeletal 13-like (LOC106587764), transcript variant X1, mRNA	KRT13
XM_014179844.1	PREDICTED: Salmo salar collagen alpha-1(I) chain-like (LOC106589632), mRNA	COL1A1
XM_014178510.1	PREDICTED: Salmo salar collagen, type I, alpha 2 (col1a2), transcript variant X1, mRNA	COL1A2
XM_014202067.1	PREDICTED: Salmo salar ictacalcin-like (LOC106606010), transcript variant X1, mRNA	
NM_001123525.1	Salmo salar beta actin (LOC100136352), mRNA	ACTB
XM_014192569.1	PREDICTED: Salmo salar collagen alpha-1(I) chain-like (LOC106600852), mRNA	COL1A1
XR_001327356.1	/	
XM_014144262.1	PREDICTED: Salmo salar collagen alpha-1(X) chain-like (LOC106571324), mRNA	COL10A1
XM_014204457.1	PREDICTED: Salmo salar collagen alpha-1(I) chain (LOC100286406), mRNA	COL1A1
XM_014209886.1	PREDICTED: Salmo salar collagen alpha-1(I) chain-like (LOC106610502), mRNA	COL1A1
XM_014142822.1	PREDICTED: Salmo salar collagen alpha-2(I) chain (LOC106570460), transcript variant X1, mRNA	COL1A2
XM_014154655.1	PREDICTED: Salmo salar collagen, type XVII, alpha 1 (col17a1), mRNA	COL17A1
XM_014131515.1	PREDICTED: Salmo salar keratin, type I cytoskeletal 13-like (LOC106564959), mRNA	KRT13
XM_014157118.1	PREDICTED: Salmo salar lipocalin-like (LOC106578378), mRNA	
XR_001321354.1	/	
XM_014131518.1	PREDICTED: Salmo salar keratin, type I cytoskeletal 13 (LOC106564961), mRNA	KRT13
XM_014201580.1	PREDICTED: Salmo salar apolipoprotein Eb-like (LOC106605689), mRNA	APOEB
XM_014141923.1	PREDICTED: Salmo salar elongation factor 1-alpha, oocyte form (LOC100136485), mRNA	EEF1AO
NM_001139607.1	Salmo salar heat shock protein 8 (hspa8), mRNA	HSC71
XM_014176433.1	PREDICTED: Salmo salar keratin, type I cytoskeletal 13-like (LOC106587783), mRNA	KRT13
NM_001123544.1	Salmo salar hyperosmotic glycine rich protein (LOC100136385), mRNA	CIRBP-B
XM_014204996.1	PREDICTED: Salmo salar collagen alpha-1(X) chain-like (LOC106607727), mRNA	COL10A1
XM_014185382.1	PREDICTED: Salmo salar ictacalcin-like (LOC106594007), transcript variant X1, mRNA	
XM_014198569.1	PREDICTED: Salmo salar neuroblast differentiation-associated protein AHNAK-like (LOC106604153), mRNA	AHNAK
XM_014176502.1	PREDICTED: Salmo salar keratin, type I cytoskeletal 13-like (LOC106587847), mRNA	KRT13

XR_001321537.1	PREDICTED: Salmo salar uncharacterized LOC106574408 (LOC106574408), ncRNA	
XM_014131516.1	PREDICTED: Salmo salar keratin, type I cytoskeletal 13-like (LOC106564960), transcript variant X1, mRNA	KRT13
XM_014140963.1	PREDICTED: Salmo salar elongation factor 2-like (LOC106569524), mRNA	EEF2
XM_014213254.1	PREDICTED: Salmo salar SPARC-like (LOC106612255), mRNA	SPARC
XM_014199897.1	PREDICTED: Salmo salar H-2 class II histocompatibility antigen gamma chain-like (LOC106604844), transcript variant X1, mRNA	CD74
XM_014177344.1	PREDICTED: Salmo salar class I histocompatibility antigen, F10 alpha chain-like (LOC106588401), mRNA	
XM_014123264.1	PREDICTED: Salmo salar desmoglein-2-like (LOC106560387), transcript variant X1, mRNA	DSG2
XM_014198623.1	PREDICTED: Salmo salar SPARC-like (LOC106604192), mRNA	SPARC
XM_014209803.1	PREDICTED: Salmo salar semaphorin-4F-like (LOC106610434), mRNA	SEMA4F
NM_001123532.1	Salmo salar heat shock protein hsp90 beta (hsp90b), mRNA	HSP90AB1
XM_014169117.1	PREDICTED: Salmo salar gelsolin-like (LOC106584171), transcript variant X1, mRNA	GSN
XM_014213290.1	PREDICTED: Salmo salar neuroblast differentiation-associated protein AHNAK-like (LOC106612274), mRNA	AHNAK
XM_014143857.1	PREDICTED: Salmo salar papilin-like (LOC106571140), transcript variant X1, mRNA	PAPLN
XM_014214349.1	PREDICTED: Salmo salar claudin-4-like (LOC106612824), mRNA	CLDN4
XM_014173892.1	PREDICTED: Salmo salar fruit protein pKIWI501-like (LOC106586507), mRNA	CD99L2
NM_001123629.1	Salmo salar elongation factor 1 alpha (LOC100136525), mRNA	EEF1AO
XR_001323464.1	/	
XM_014140215.1	PREDICTED: Salmo salar desmoplakin-like (LOC106569165), transcript variant X1, mRNA	DSP
XR_001328652.1	PREDICTED: Salmo salar uncharacterized LOC106604326 (LOC106604326), ncRNA	
XM_014176441.1	PREDICTED: Salmo salar keratin, type I cytoskeletal 13-like (LOC106587793), mRNA	KRT13
XM_014188776.1	/	
XM_014192087.1	PREDICTED: Salmo salar ornithine decarboxylase 1 (odc1), mRNA	ODC1-A
XM_014146179.1	PREDICTED: Salmo salar nuclease-sensitive element-binding protein 1-like (LOC106572208), transcript variant X1, mRNA	YBX1
