# Supplementary material for: Comparative transcriptome analysis of trout skin pigment cells
Source: BMC Genomics. 2019 May 9;20:359. doi: 10.1186/s12864-019-5714-1 (PMC6509846; doi:10.1186/s12864-019-5714-1)
Supplement: Supplementary file 4 — Table S4. 100 transcripts with the highest fold change between marble and brown trout samples. (PDF 108 kb) [file 12864_2019_5714_MOESM4_ESM.pdf]

Table S4

100 transcripts with the highest fold change between marble and brown trout samples.

Transcript	Annotation	Gene symbol	Fold change
XR_001328906.1	XR_001328906.1		437.79
XM_014147610.1	PREDICTED: Salmo salar pleckstrin homology domain-containing family A member 6-like (LOC106573014), mRNA	PLEKHA6	433.69
XM_014187257.1	PREDICTED: Salmo salar uncharacterized LOC106595915 (LOC106595915), partial mRNA	ERVPABLB-1	358.54
XM_014167198.1	PREDICTED: Salmo salar heterogeneous nuclear ribonucleoprotein A1 (hnrnpa1), mRNA	HNRNPA1	316.66
XM_014142551.1	XM_014142551.1		283.97
XM_014123307.1	PREDICTED: Salmo salar pituitary adenylate cyclase-activating polypeptide type I receptor-like (LOC106560417), mRNA	ADCYAP1R1	270.19
XR_001322257.1	/		246.69
XR_001326720.1	/		238.79
XR_001323502.1	/		227.03
XR_001324509.1	PREDICTED: Salmo salar uncharacterized LOC106587838 (LOC106587838), ncRNA	ENOD2	221.08
XM_014215678.1	PREDICTED: Salmo salar GTPase IMAP family member 4-like (LOC106613446), transcript variant X1, mRNA	GIMAP4	205.69
XM_014188858.1	/		205.04
XM_014176304.1	PREDICTED: Salmo salar uncharacterized LOC106587709 (LOC106587709), partial mRNA	ENOD2	200.19
XM_014186015.1	/		183.95
XM_014158647.1	PREDICTED: Salmo salar MAGUK p55 subfamily member 3-like (LOC106579097), transcript variant X1, mRNA	MPP3	141.91
XR_001318647.1	/		138.01
XR_001325260.1	PREDICTED: Salmo salar uncharacterized LOC106591266 (LOC106591266), ncRNA		130.43
XM_014186896.1	PREDICTED: Salmo salar GTPase IMAP family member 4-like (LOC106595525), partial mRNA	GIMAP4	124.99
XM_014216093.1	PREDICTED: Salmo salar phosphatidylinositol 3-kinase regulatory subunit gamma-like (LOC106613635), mRNA	PIK3R1	121.89
XM_014124464.1	PREDICTED: Salmo salar Mov10 RISC complex RNA helicase like 1 (mov10l1), transcript variant X1, mRNA	MOV10L1	112.81
XM_014132388.1	PREDICTED: Salmo salar myosin heavy chain, fast skeletal muscle-like (LOC106565368), mRNA		107.58
XM_014185696.1	PREDICTED: Salmo salar uncharacterized LOC106594338 (LOC106594338), transcript variant X1, mRNA		103.64
XM_014173425.1	PREDICTED: Salmo salar parathyroid hormone 2 receptor-like (LOC106586291), mRNA	PTH2R	103.39
XR_001326520.1	PREDICTED: Salmo salar uncharacterized LOC106595957 (LOC106595957), misc_RNA	ZP2	101.68
XR_001327654.1	PREDICTED: Salmo salar uncharacterized LOC106600012 (LOC106600012), transcript variant X1, ncRNA	SGS3	100.55
XM_014205953.1	PREDICTED: Salmo salar gap junction delta-2 protein-like (LOC106608171), mRNA	GJD2	100.51
XM_014190851.1	PREDICTED: Salmo salar chloride channel protein 2-like (LOC106599558), mRNA	CLCN2	100.36

XM_014198602.1	PREDICTED: Salmo salar cationic amino acid transporter 2-like (LOC106604180), transcript variant X1, mRNA	SLC7A2	99.12
XR_001328209.1	/		95.63
XM_014142841.1	/		93.22
XM_014149368.1	PREDICTED: Salmo salar chromosome ssa16 open reading frame, human C19orf45 (cssa16h19orf45), transcript variant X1, mRNA	C19ORF45	91.4
XM_014190098.1	PREDICTED: Salmo salar calsyntenin-2-like (LOC106599045), transcript variant X1, mRNA	CLSTN2	90.19
NM_001140859.2	Salmo salar Ecto-ADP-ribosyltransferase 4 (nar4), mRNA	ART4	87.39
XM_014186199.1	PREDICTED: Salmo salar solute carrier organic anion transporter family member 1C1-like (LOC106594814), mRNA	SLCO1C1	86.38
XM_014136246.1	PREDICTED: Salmo salar glucose-induced degradation protein 8 homolog (LOC106567219), transcript variant X1, mRNA	GID8	81.73
XM_014163718.1	PREDICTED: Salmo salar F-box protein 40 (fbxo40), transcript variant X1, mRNA	FBXO40	81.69
XM_014155408.1	PREDICTED: Salmo salar formin-2-like (LOC106577437), mRNA	FMN2	81.43
XR_001326680.1	XR_001326680.1		80.55
XM_014200492.1	PREDICTED: Salmo salar myomesin-3-like (LOC106605141), mRNA		79.84
XM_014160998.1	PREDICTED: Salmo salar HERV-H LTR-associating 1 (hhla1), transcript variant X1, mRNA	HHLA1	77.83
XM_014138419.1	PREDICTED: Salmo salar ankyrin repeat and LEM domain-containing protein 2-like (LOC106568248), mRNA	ANKLE2	76.72
XM_014168507.1	PREDICTED: Salmo salar exportin-6-like (LOC106583883), mRNA	XPO6	76.16
XM_014135835.1	PREDICTED: Salmo salar dihydropyridine-sensitive L-type skeletal muscle calcium channel subunit alpha-1-like (LOC106567031), transcript variant X1, mRNA		75.40
XR_001318609.1	/		73.3
XR_001327406.1	/		71.74
XM_014208304.1	PREDICTED: Salmo salar amidohydrolase domain containing 1 (amdhd1), mRNA	AMDHD1	71.67
XM_014176586.1	PREDICTED: Salmo salar immunoglobulin superfamily containing leucine-rich repeat protein 2-like (LOC106587927), mRNA	ISLR2	71.22
XM_014146039.1	PREDICTED: Salmo salar WAP four-disulfide core domain protein 2-like (LOC106572147), mRNA		68.1
XM_014124661.1	PREDICTED: Salmo salar DENN/MADD domain containing 5A (dennd5a), transcript variant X1, mRNA	DENND5B	66.56
XR_001330121.1	/		63.84
XM_014146040.1	PREDICTED: Salmo salar perlwapin-like (LOC106572148), mRNA	WFDC18	61.83
XM_014189481.1	PREDICTED: Salmo salar zona pellucida sperm-binding protein 2-like (LOC106598441), partial mRNA	ZP2	61.61
XM_014168207.1	PREDICTED: Salmo salar glycerol-3-phosphate dehydrogenase [NAD(+)], cytoplasmic-like (LOC106583703), transcript variant X1, mRNA	GPD1	60.77
NM_001141474.1	Salmo salar Perlwapin (pwap), mRNA		60.24
XM_014191612.1	PREDICTED: Salmo salar methyltransferase-like protein 25 (LOC106600310), transcript variant X1, mRNA	METTL25	59.45

XM_014139473.1	PREDICTED: Salmo salar autoimmune regulator (aire), transcript variant X1, mRNA	AIRE	59.22
XM_014205412.1	PREDICTED: Salmo salar cGMP-dependent protein kinase 1-like (LOC106607930), mRNA	PRKG2	58.79
XR_001322800.1	/		58.29
XM_014173424.1	PREDICTED: Salmo salar potassium voltage-gated channel subfamily C member 2-like (LOC106586287), partial mRNA	KCNC2	56.4
XM_014191903.1	PREDICTED: Salmo salar polyhomeotic homolog 3 (Drosophila) (phc3), transcript variant X1, mRNA	PHC3	55.72
XR_001321655.1	/		55.66
XM_014138508.1	PREDICTED: Salmo salar rabphilin-3A-like (LOC106568299), mRNA	RPH3A	54.42
XM_014146041.1	PREDICTED: Salmo salar WAP four-disulfide core domain protein 18-like (LOC106572149), mRNA	WFDC2	53.93
XM_014129326.1	PREDICTED: Salmo salar cationic amino acid transporter 2-like (LOC106563607), transcript variant X1, mRNA	SLC7A2	52.49
XM_014189578.1	PREDICTED: Salmo salar zona pellucida sperm-binding protein 4-like (LOC106598538), partial mRNA	ZP2	51.47
XM_014204514.1	/		51.21
XR_001328768.1	/		50.05
XR_001330446.1	PREDICTED: Salmo salar uncharacterized LOC106613127 (LOC106613127), ncRNA	CPY1	48.6
XM_014123745.1	PREDICTED: Salmo salar allantoinase, mitochondrial-like (LOC106560661), mRNA	ALN	48.56
XM_014131709.1	PREDICTED: Salmo salar 1,25-dihydroxyvitamin D(3) 24-hydroxylase, mitochondrial-like (LOC106565076), mRNA	CYP24A1	48.18
XR_001319244.1	/		48.1
XM_014189628.1	/		46.56
XM_014204641.1	PREDICTED: Salmo salar zona pellucida sperm-binding protein 4-like (LOC106607570), mRNA	ZP2	46.14
XM_014183743.1	PREDICTED: Salmo salar GTPase IMAP family member 7-like (LOC106592401), partial mRNA	GIMAP7	45.82
XM_014129971.1	PREDICTED: Salmo salar zinc finger protein GLIS3-like (LOC106564006), transcript variant X1, mRNA	GLIS3	43.82
XM_014163411.1	/		43.33
XM_014125534.1	PREDICTED: Salmo salar homeobox protein DBX1-B-like (LOC106561495), mRNA	DBX1B	43.03
XR_001327935.1	/		42.69
XM_014178546.1	PREDICTED: Salmo salar zinc transporter ZIP4-like (LOC106588961), mRNA	SLC39A4	42.51
XR_001320475.1	/		42.37
XR_001326333.1	/		41.83
XR_001324616.1	/		41.38
XR_001325688.1	/		40.85
XM_014200192.1	PREDICTED: Salmo salar M-protein, striated muscle-like (LOC106605001), mRNA		40.78
XM_014190493.1	PREDICTED: Salmo salar zona pellucida sperm-binding protein 4-like (LOC106599323), partial mRNA	ZP4	40.65
XM_014191555.1	PREDICTED: Salmo salar uncharacterized LOC106600238 (LOC106600238), partial mRNA	ROBO2	39.97
XR_001326139.1	/		38.95
XM_014125050.1	PREDICTED: Salmo salar uncharacterized LOC106561275 (LOC106561275), mRNA		38.9
XM_014187962.1	PREDICTED: Salmo salar collectin-10-like (LOC106596689), transcript variant X1, mRNA	COLEC10	38.64

XM_014205400.1	PREDICTED: Salmo salar fibroblast growth factor-binding protein 2-like (LOC106607923), mRNA	FGFBP2	38.59
XM_014187921.1	PREDICTED: Salmo salar collectin-10-like (LOC106596649), transcript variant X1, mRNA	COLEC10	38.56
XM_014187183.1	PREDICTED: Salmo salar stonustoxin subunit beta-like (LOC106595837), mRNA		38.17
XR_001328959.1	PREDICTED: Salmo salar uncharacterized LOC106605903 (LOC106605903), ncRNA	NUP210	37.7
XR_001326698.1	PREDICTED: Salmo salar uncharacterized LOC106596535 (LOC106596535), transcript variant X1, ncRNA	DFNA5	37.67
NM_001173862.1	Salmo salar Solute carrier family 35 member F4 (s35f4), mRNA	SLC35F4	37.66
XM_014139950.1	PREDICTED: Salmo salar otolin-1 (LOC106569022), mRNA	OTOL1	37.65
XM_014206438.1	/		37.59
XM_014166253.1	PREDICTED: Salmo salar glutamate receptor U1-like (LOC106582807), transcript variant X1, mRNA	KBP	37.33
XM_014194922.1	PREDICTED: Salmo salar FRAS1-related extracellular matrix protein 2-like (LOC106602345), partial mRNA	FREM2	36.51
XM_014152733.1	PREDICTED: Salmo salar solute carrier organic anion transporter family member 1C1-like (LOC106575962), mRNA	SLCO1C1	36.36
