# Supplementary material for: Comparative transcriptome analysis of trout skin pigment cells
Source: BMC Genomics. 2019 May 9;20:359. doi: 10.1186/s12864-019-5714-1 (PMC6509846; doi:10.1186/s12864-019-5714-1)
Supplement: Supplementary file 5 — Table S5. 100 transcripts with the highest fold change between brown and marble trout samples. (PDF 111 kb) [file 12864_2019_5714_MOESM5_ESM.pdf]

Table S5

100 transcripts with the highest fold change between brown and marble trout samples.

Transcript	Annotation	Gene symbol	Fold change
XR_001318747.1	/		15591.21
XM_014209865.1	PREDICTED: Salmo salar up-regulator of cell proliferation-like (LOC106610488), mRNA	GVIN1	5220.76
XM_014149618.1	PREDICTED: Salmo salar histone H1, orphon-like (LOC106574076), mRNA	KRTAP4-9	1639.99
XM_014184266.1	/		1604.34
XM_014183069.1	/		1585.70
XM_014153852.1	PREDICTED: Salmo salar fish-egg lectin-like (LOC106576607), mRNA		1574.55
XM_014153850.1	PREDICTED: Salmo salar fish-egg lectin-like (LOC106576606), mRNA		1276.44
XM_014184132.1	/		1269.68
XM_014152427.1	PREDICTED: Salmo salar coagulation factor VII-like (LOC106575751), partial mRNA	F7	707.77
XM_014176158.1	PREDICTED: Salmo salar aggrecan core protein-like (LOC106587597), mRNA	ACAN	686.26
XM_014156826.1	PREDICTED: Salmo salar protein phosphatase methylesterase 1-like (LOC106578192), mRNA	PPME1	632.67
XM_014127456.1	PREDICTED: Salmo salar aggrecan core protein-like (LOC106562525), partial mRNA	ACAN	509.89
XM_014135351.1	PREDICTED: Salmo salar succinate dehydrogenase [ubiquinone] iron-sulfur subunit, mitochondrial-like (LOC106566856), mRNA	SDHB	496.94
XM_014182108.1	PREDICTED: Salmo salar fish-egg lectin-like (LOC106590936), mRNA		468.49
XM_014127310.1	PREDICTED: Salmo salar aggrecan core protein (LOC100380783), mRNA	ACAN	466.31
XM_014129439.1	PREDICTED: Salmo salar ependymin-like (LOC106563665), mRNA	EPD	451.16
XM_014208593.1	PREDICTED: Salmo salar uncharacterized LOC106609620 (LOC106609620), mRNA	SLAMF9	363.07
XM_014190503.1	PREDICTED: Salmo salar fish-egg lectin-like (LOC106599329), mRNA		340.95
XM_014205465.1	PREDICTED: Salmo salar spermatogenesis-associated protein 7-like (LOC106607950), transcript variant X1, mRNA	SPATA7	336.39
XM_014212430.1	PREDICTED: Salmo salar protocadherin gamma-C3-like (LOC106611830), mRNA	PCDHAC2	300.23
XR_001324659.1	/		297.88
XR_001324660.1	/		263.95
XM_014141362.1	PREDICTED: Salmo salar tektin 2 (testicular) (tekt2), transcript variant X1, mRNA	TEKT2	263.26
XM_014187544.1	PREDICTED: Salmo salar endonuclease domain-containing 1 protein-like (LOC106596234), mRNA	ENDOD1	246.15
XM_014176173.1	PREDICTED: Salmo salar brevican core protein-like (LOC106587613), partial mRNA	ACAN	219.07
XM_014129864.1	PREDICTED: Salmo salar protocadherin Fat 3-like (LOC106563919), partial mRNA	FAT3	216.32
XM_014204681.1	PREDICTED: Salmo salar potassium channel, two pore domain subfamily K, member 3 (kcnk3), mRNA	KCNK3	208.10
NM_001140986.1	Salmo salar Mannose-specific lectin (asal), mRNA	LECASAL	192.28
XM_014209598.1	PREDICTED: Salmo salar hydroperoxide isomerase ALOXE3-like (LOC106610314), mRNA	ALOXE3	186.55

XM_014143850.1	PREDICTED: Salmo salar complement C1q-like protein 2 (LOC106571137), mRNA	C1QL2	183.44
XM_014215093.1	PREDICTED: Salmo salar calsyntenin-2-like (LOC106613134), mRNA	CLSTN2	170.32
XM_014189296.1	PREDICTED: Salmo salar fish-egg lectin-like (LOC106598243), mRNA		159.68
XM_014158126.1	PREDICTED: Salmo salar snaclec 1-like (LOC106578890), mRNA	CLEC4M	154.81
XM_014123350.1	PREDICTED: Salmo salar fibronectin type III domain containing 7 (fndc7), mRNA	FNDC7	148.43
XM_014143884.1	PREDICTED: Salmo salar complement C1q-like protein 2 (LOC106571149), partial mRNA	C1QL2	146.73
XM_014143400.1	PREDICTED: Salmo salar D-beta-hydroxybutyrate dehydrogenase, mitochondrial-like (LOC106570820), partial mRNA	BDH1	145.96
XM_014141283.1	PREDICTED: Salmo salar solute carrier family 1 (glutamate transporter), member 7 (slc1a7), partial mRNA	SLC1A7	144.33
XM_014163567.1	PREDICTED: Salmo salar P2Y purinoceptor 13-like (LOC106581492), mRNA	P2RY13	138.00
XM_014170041.1	PREDICTED: Salmo salar ATP-binding cassette sub-family C member 8-like (LOC106584588), transcript variant X1, mRNA	ABCC8	136.99
NM_001204894.1	Salmo salar SCARB1-like protein 2 (LOC100534606), mRNA	SCARB1	134.98
XM_014132251.1	/		134.03
XM_014194850.1	PREDICTED: Salmo salar tetratricopeptide repeat protein 39B-like (LOC106602306), transcript variant X1, mRNA	TTC39B	131.59
XM_014199820.1	PREDICTED: Salmo salar protein bicaudal C homolog 1-A-like (LOC106604787), transcript variant X1, mRNA	BICC1	127.37
XM_014145307.1	PREDICTED: Salmo salar OTU domain-containing protein 5-A-like (LOC106571839), transcript variant X1, mRNA	OTUD5A	126.03
XM_014141364.1	PREDICTED: Salmo salar tektin 2 (testicular) (tekt2), transcript variant X3, mRNA	TEKT2	123.14
XM_014125955.1	PREDICTED: Salmo salar lipoma HMGIC fusion partner-like 3 protein (LOC106561734), transcript variant X1, mRNA	LHFPL3	118.51
XR_001319832.1	/		115.60
XM_014176556.1	PREDICTED: Salmo salar involucrin-like (LOC106587898), mRNA	ORF73	114.47
XM_014194222.1	PREDICTED: Salmo salar parvalbumin, thymic CPV3-like (LOC106601824), mRNA		114.15
XM_014156517.1	PREDICTED: Salmo salar FH1/FH2 domain-containing protein 1-like (LOC106578003), mRNA	FHOD1	112.23
NM_001140812.1	Salmo salar Fish-egg lectin (fel), mRNA		104.28
XM_014187719.1	PREDICTED: Salmo salar endonuclease domain-containing 1 protein-like (LOC106596433), mRNA	ENDOD1	102.77
XM_014137863.1	PREDICTED: Salmo salar diacylglycerol O-acyltransferase 2-like (LOC106567948), mRNA	DGAT2	101.97
XM_014135840.1	PREDICTED: Salmo salar complement C1q-like protein 4 (LOC106567034), transcript variant X1, mRNA	C1QL4	99.73
XM_014129902.1	PREDICTED: Salmo salar aldolase C, fructose-bisphosphate (aldoc), mRNA	ALDOCB	98.82
XM_014209575.1	PREDICTED: Salmo salar tetratricopeptide repeat protein 39B-like (LOC106610300), transcript variant X1, mRNA	TTC39B	98.33
XM_014192725.1	PREDICTED: Salmo salar heme-binding protein 2-like (LOC106600932), mRNA	HEBP2	97.11

XM_014201005.1	PREDICTED: Salmo salar uncharacterized LOC106605403 (LOC106605403), mRNA	MMP2	95.42
XR_001321185.1	PREDICTED: Salmo salar uncharacterized LOC106572770 (LOC106572770), ncRNA	TNFRSF14	91.79
XM_014155145.1	PREDICTED: Salmo salar catechol O-methyltransferase domain-containing protein 1-like (LOC106577229), mRNA	COMTD1	90.79
XM_014182058.1	PREDICTED: Salmo salar protocadherin alpha-C2-like (LOC106590882), transcript variant X1, mRNA	PCDHAC2	90.52
XM_014195783.1	PREDICTED: Salmo salar solute carrier family 13 member 2-like (LOC106602849), mRNA	SLC13A2	90.05
XM_014181646.1	PREDICTED: Salmo salar lipocalin-like (LOC106590538), mRNA		89.03
XM_014209660.1	PREDICTED: Salmo salar tetratricopeptide repeat protein 39B-like (LOC106610345), transcript variant X1, mRNA	TTC39B	88.04
XM_014196511.1	PREDICTED: Salmo salar seipin-like (LOC106603188), transcript variant X1, mRNA	BSCL2	78.69
XM_014133861.1	PREDICTED: Salmo salar cytochrome b ascorbate-dependent protein 3-like (LOC106566050), partial mRNA	CYB561A3	78.00
XM_014139983.1	PREDICTED: Salmo salar alcohol dehydrogenase, iron containing, 1 (adhfe1), transcript variant X1, mRNA	ADHFE1	76.49
XM_014177900.1	PREDICTED: Salmo salar leucine-rich repeat-containing protein 3B-like (LOC106588675), transcript variant X1, mRNA	LRRC3B	76.27
NM_001159373.1	Salmo salar leukolectin protein (ll), mRNA		75.61
XM_014183806.1	PREDICTED: Salmo salar pyruvate carboxylase, mitochondrial-like (LOC106592454), mRNA	PC	74.49
XM_014147535.1	PREDICTED: Salmo salar PDZ domain-containing protein 4-like (LOC106572942), mRNA	PDZD4	73.36
XR_001319241.1	/		71.95
XM_014196679.1	PREDICTED: Salmo salar fish-egg lectin-like (LOC106603265), mRNA		71.68
XM_014158988.1	PREDICTED: Salmo salar ATP-sensitive inward rectifier potassium channel 12-like (LOC106579252), mRNA	KCNJ12	71.62
XM_014186237.1	PREDICTED: Salmo salar fibronectin-like (LOC106594853), partial mRNA	FNDC7	69.80
XM_014187828.1	PREDICTED: Salmo salar patatin-like phospholipase domain-containing protein 2 (LOC106596546), mRNA	PNPLA2	69.75
XM_014215138.1	PREDICTED: Salmo salar protein NLRC3-like (LOC106613151), mRNA	NLRC3	69.20
XM_014127148.1	PREDICTED: Salmo salar calcitonin-1 (LOC106562334), transcript variant X1, mRNA		68.41
XM_014185949.1	PREDICTED: Salmo salar perforin-1-like (LOC106594578), mRNA	PRF1	67.99
XM_014199226.1	PREDICTED: Salmo salar relaxin receptor 1-like (LOC106604530), mRNA	RXFP1	66.99
XM_014173051.1	PREDICTED: Salmo salar rac GTPase-activating protein 1-like (LOC106586148), transcript variant X1, mRNA	RACGAP1	66.83
XM_014194844.1	PREDICTED: Salmo salar tetratricopeptide repeat protein 39B-like (LOC106602300), transcript variant X1, mRNA	TTC39B	65.56
NM_001141004.1	Salmo salar Guanine nucleotide-binding protein GI/GS/GO subunit gamma-T2 (gbgt2), mRNA	GNGT2	64.31
XM_014211418.1	PREDICTED: Salmo salar otoferlin-like (LOC106611328), transcript variant X1, mRNA	OTOF	64.00
XM_014183359.1	PREDICTED: Salmo salar cartilage acidic protein 1-like (LOC106592054), mRNA	CRTAC1	63.56

XM_014162432.1	PREDICTED: Salmo salar complement C1q-like protein 4 (LOC106580900), transcript variant X1, mRNA	U88	62.63
NM_001165272.1	Salmo salar Beta-2-glycoprotein 1 (apoh), mRNA	APOH	62.56
XM_014145744.1	PREDICTED: Salmo salar plasma membrane calcium-transporting ATPase 3-like (LOC106572032), transcript variant X1, mRNA	ATP2B3	61.33
XM_014170518.1	PREDICTED: Salmo salar cytoskeleton-associated protein 2-like (LOC106584893), transcript variant X1, mRNA	CKAP2L	60.96
XM_014147229.1	PREDICTED: Salmo salar lipoma HMGIC fusion partner-like 4 protein (LOC106572763), transcript variant X1, mRNA	LHFPL4	60.29
XM_014203003.1	PREDICTED: Salmo salar parvalbumin, thymic CPV3-like (LOC106606664), mRNA		60.26
NM_001140514.1	Salmo salar Abhydrolase domain-containing protein 12 (abd12), mRNA	ABHD12	59.44
XM_014188215.1	PREDICTED: Salmo salar cytolysin Src-1-like (LOC106596976), transcript variant X1, mRNA		58.93
XR_001323291.1	/		58.17
XM_014183938.1	PREDICTED: Salmo salar perforin-1-like (LOC106592600), partial mRNA	PRF1	57.71
XM_014153849.1	PREDICTED: Salmo salar fish-egg lectin-like (LOC106576605), mRNA		57.17
XM_014203024.1	PREDICTED: Salmo salar sterile alpha motif domain-containing protein 9-like (LOC106606682), mRNA	SAMD9L	56.97
XM_014148186.1	PREDICTED: Salmo salar synaptotagmin-9-like (LOC106573278), mRNA	SYT9	55.77
XM_014145754.1	PREDICTED: Salmo salar deleted in malignant brain tumors 1 protein-like (LOC106572034), transcript variant X1, mRNA	DMBT1	54.01
XM_014181632.1	PREDICTED: Salmo salar lipocalin-like (LOC106590529), mRNA		53.94
