# Supplementary material for: Comparative transcriptome analysis of trout skin pigment cells
Source: BMC Genomics. 2019 May 9;20:359. doi: 10.1186/s12864-019-5714-1 (PMC6509846; doi:10.1186/s12864-019-5714-1)
Supplement: Supplementary file 6 — Figure S6. Gene expression patterns of four candidate genes in differently pigmented regions in the skin of hybrids. Red, red spot; dark brown, dark region; light brown, light region. (PDF 345 kb) [file 12864_2019_5714_MOESM6_ESM.pdf]

Figure S6

Gene expression patterns of four candidate genes in differently pigmented regions in the skin of hybrids. Red, red spot; dark brown, dark region; light brown, light region.

cdkn1a

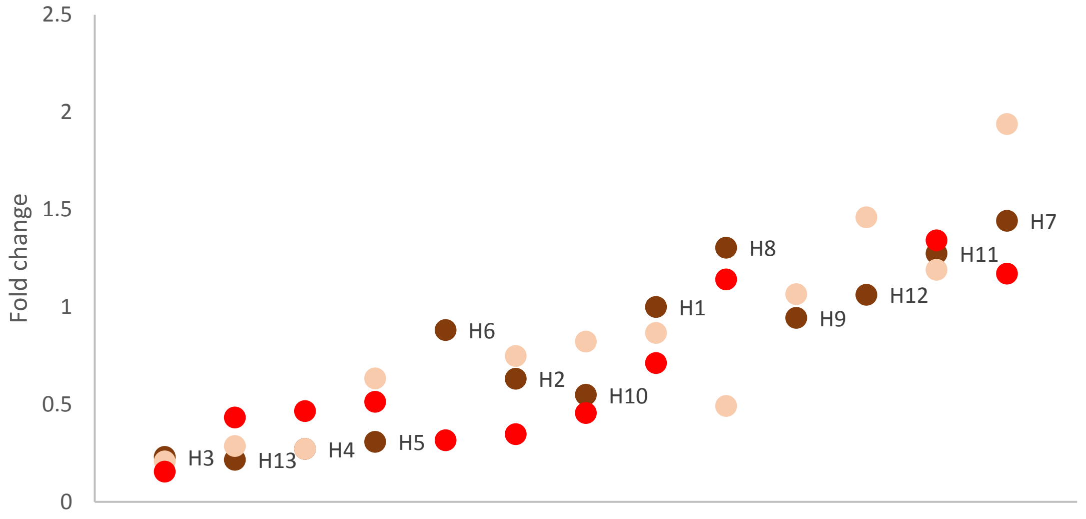

tjp1

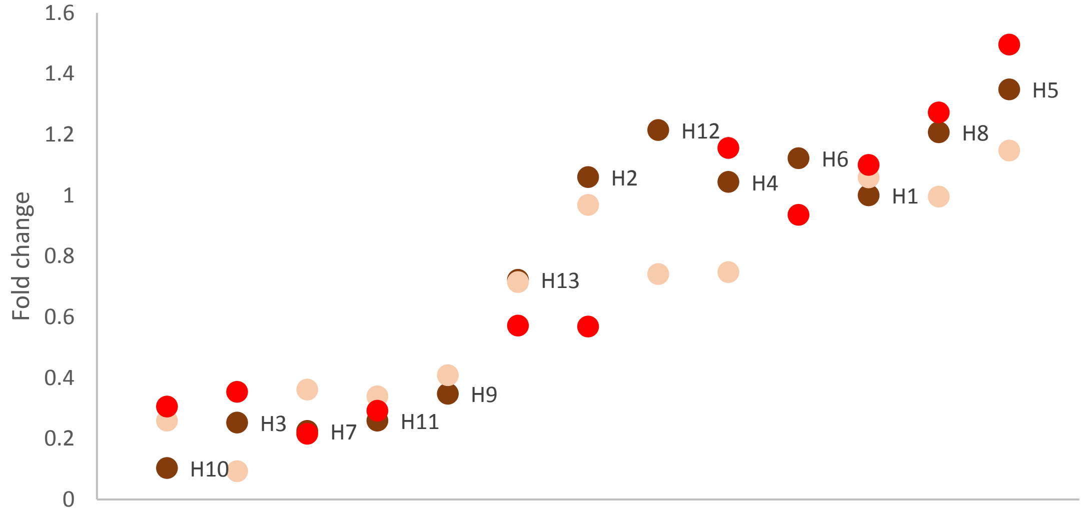

clcn2

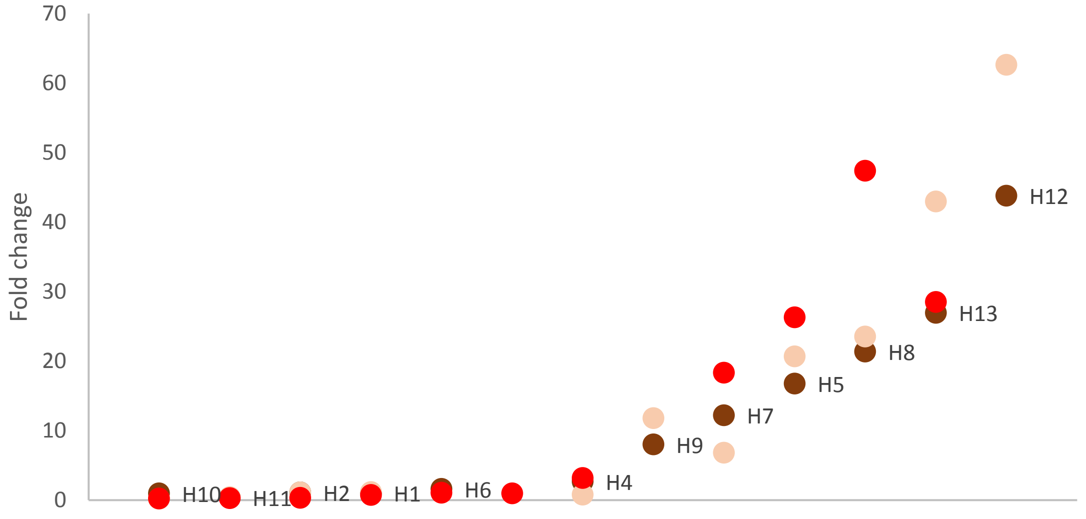

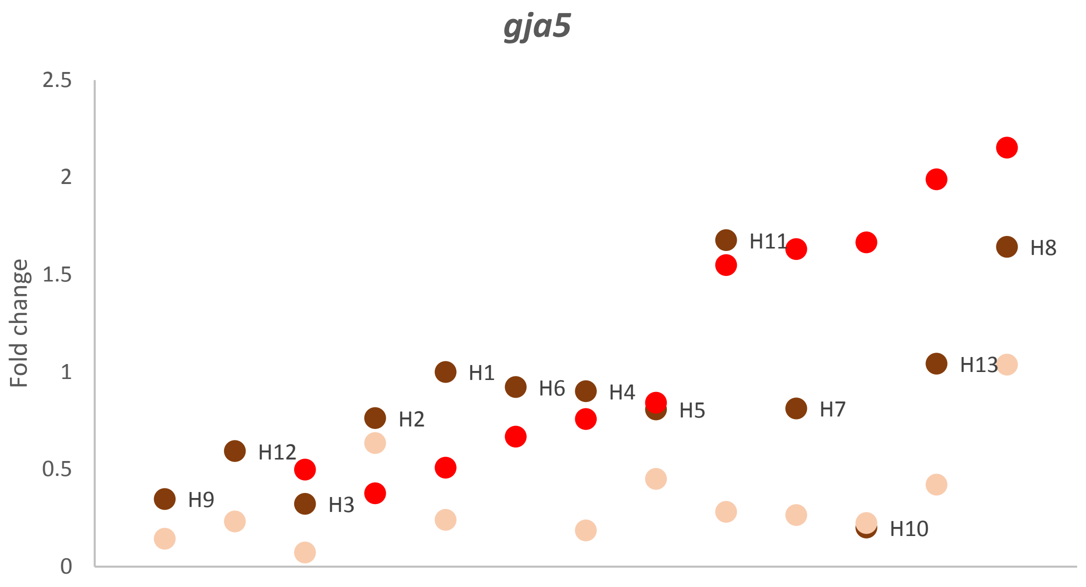

gja5
Fold change
2.5
2
1.5
1
0.5
0
H11
H8
H13
H1
H6
H4
H5
H7
H2
H12
H9
H3
H10
