# Supplementary material for: Comparative transcriptome analysis of trout skin pigment cells
Source: BMC Genomics. 2019 May 9;20:359. doi: 10.1186/s12864-019-5714-1 (PMC6509846; doi:10.1186/s12864-019-5714-1)
Supplement: Supplementary file 7 — Table S7.. Level of brown trout introgression in hybrid individuals. (PDF 417 kb) [file 12864_2019_5714_MOESM7_ESM.pdf]

Table S7

Level of brown trout introgression into marble trout.

Hybrids	Brown trout introgression (%)
H3	57,1
H4	53,6
H1	50
H2	50
H6	46,4
H5	42,9
H7	39,3
H11	35,7
H8	32,1
H13	21,4
H9	15,4
H10	14,3
H12	10,7
