# Supplementary material for: Comparative transcriptome analysis of trout skin pigment cells
Source: BMC Genomics. 2019 May 9;20:359. doi: 10.1186/s12864-019-5714-1 (PMC6509846; doi:10.1186/s12864-019-5714-1)

Figure S8

Correlation between the expression of gja5 gene in the red spots of hybrids and overall colour tone of the skin in hybrids.

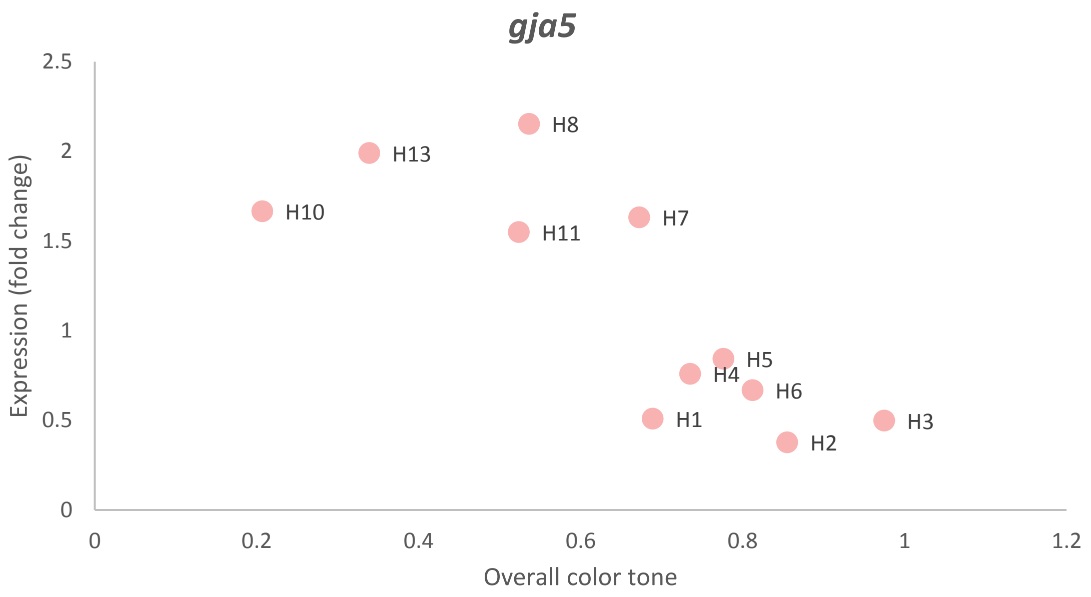

Supplement: Supplementary file 8 — Figure S8. Correlation between the expression of gja5 gene in the red spots of hybrids and overall colour tone of the skin in hybrids. (PDF 315 kb) [file 12864_2019_5714_MOESM8_ESM.pdf]
